# Genomic prediction and allele mining of agronomic and morphological traits in pea (*Pisum sativum*) germplasm collections

**DOI:** 10.3389/fpls.2023.1320506

**Published:** 2023-12-22

**Authors:** Margherita Crosta, Massimo Romani, Nelson Nazzicari, Barbara Ferrari, Paolo Annicchiarico

**Affiliations:** ^1^Council for Agricultural Research and Economics (CREA), Research Centre for Animal Production and Aquaculture, Lodi, Italy; ^2^Department of Sustainable Crop Production, Catholic University of Sacred Heart, Piacenza, Italy

**Keywords:** crop quality, drought tolerance, genetic structure, genomic selection, grain yield, grain protein content, molecular distinctness, trait genetic architecture

## Abstract

Well-performing genomic prediction (GP) models for polygenic traits and molecular marker sets for oligogenic traits could be useful for identifying promising genetic resources in germplasm collections, setting core collections, and establishing molecular variety distinction. This study aimed at (i) defining GP models and key marker sets for predicting 15 agronomic or morphological traits in germplasm collections, (ii) verifying the GP model usefulness also for selection in breeding programs, (iii) investigating the consistency between molecular and phenotypic diversity patterns, and (iv) identifying genomic regions associated with to the target traits. The study was based on phenotyping data and over 41,000 genotyping-by-sequencing-generated SNP markers of 220 landraces or old cultivars belonging to a world germplasm collection and 11 modern cultivars. Non-metric multi-dimensional scaling (NMDS) and an analysis of population genetic structure indicated a high level of genetic differentiation of material from Western Asia, a major West-East diversity gradient, and quite limited genetic diversity of the improved germplasm. Mantel’s test revealed a low correlation (*r* = 0.12) between phenotypic and molecular diversity, which increased (*r* = 0.45) when considering only the molecular diversity relative to significant SNPs from genome-wide association analyses. These analyses identified, inter alia, several areas of chromosome 6 involved in a largely pleiotropic control of vegetative or reproductive organ pigmentation. We found various significant SNPs for grain and straw yield under severe drought and onset of flowering, and one SNP on chromosome 5 for grain protein content. GP models displayed moderately high predictive ability (0.43 to 0.61) for protein content, grain and straw yield, and onset of flowering, and high predictive ability (0.76) for individual seed weight, based on intra-population, intra-environment cross-validations. The inter-population, inter-environment assessment of the models trained on the germplasm collection for breeding material of three recombinant inbred line (RIL) populations, which was challenged by much narrower diversity of the material, over eight-fold less available markers and quite different test environments, led to an overall loss of predictive ability of about 40% for seed weight, 50% for protein content and straw yield, and 60% for onset of flowering, and no prediction for grain yield. Within-RIL population predictive ability differed among populations.

## 1 Introduction

Enhancing grain legume cultivation is of paramount importance for European agriculture to improve its sustainability in terms of soil fertility, energy efficiency, greenhouse gas emissions and crop biodiversity (Watson et al., 2017; Barbieri et al., 2021; Billen et al., 2021) and to increase its self-sufficiency for high-protein feedstuff (currently amounting to 45%). Indeed, the proportion of grain legume-cultivated area in Europe is almost ten-fold lower than that in the rest of the world (1.5% vs. 14.5%; Watson et al., 2017). Plant breeding can be pivotal, to reduce the profitability gap with cereals that limits the cultivation of legumes (Rubiales et al., 2021). Field pea (*Pisum sativum* L.) is a grain legume with high interest for cultivation in Southern and Western Europe, because of its high yielding ability compared with other cool-season grain legumes (Carrouée et al., 2003; Annicchiarico, 2008). However, its protein yield per unit of area may be lower than that of some other cool-season grain legume species, e.g., white lupin (Annicchiarico, 2008; Cernay et al., 2016), because of only moderate seed protein content, whose improvement is therefore a major breeding objective (Duc et al., 2015). While modern commercial cultivars display seed protein content on the dry matter mostly in the range of 22-24%, landrace or old cultivar material may achieve values of 27-30% (Coyne et al., 2005; Jha et al., 2015; Annicchiarico et al., 2017). Another key breeding target, especially in Southern Europe and in the context of climate change, is greater yielding ability under severe drought (Bagheri et al., 2023), another feature for which landrace germplasm could be a valuable genetic resource (Annicchiarico et al., 2017).

Pea has a long history of domestication whose first steps date back to about 9,000 years BC in the Fertile Crescent (Zohary and Hopf, 1973), with a later domestication event in Abyssinia that gave rise to *P. sativum L. subsp. abyssinicum* (presently grown in Ethiopia and Yemen) (Trněný et al., 2018; Weeden, 2018). Several ex-situ pea germplasm collections have been established with the goal of preserving the outstanding biodiversity generated during this long and geographically-diversified domestication process. They include, on the whole, over 55,000 accessions (Smýkal et al., 2008), with major collections hosted by the Vavilov Institute of Plant Genetic Resources, the Australian Temperate Field Crops Collection, and the United States Department of Agriculture (Smýkal et al., 2012). A key issue to enable the exploitation of this huge amount of genetic resources for breeding purposes is the establishment of an effective criterion for the definition of core collections, which should include a reduced number of accessions to allow phenotypic characterization at a reasonable cost while maximizing the genetic variability for traits of possible interest. The site of origin according to passport data has frequently been used as the criterion for selection of germplasm accessions to be included in core collections, in the absence of relevant morpho-physiological and agronomic information (Knüpffer and Van Hintum, 1995). The exploitation of molecular information represents an alternative to the use of passport data for the selection of accessions featuring large diversity for useful traits, considering that next-generation sequencing techniques have substantially lowered the genotyping costs (Elshire et al., 2011; Taranto et al., 2018; Singh et al., 2019). Methods based on DNA reduced-representation libraries, such as genotyping-by-sequencing (GBS), are particularly suitable for species featuring a large genome, such as pea (∼ 4.45 Gb), for which whole genome re-sequencing of a large number of individuals would hardly be affordable (Kreplak et al., 2019; Pavan et al., 2020). Pea core collections set up according to marker data have already been proposed (Jing et al., 2012; Holdsworth et al., 2017), but a prerequisite for their practical usefulness for breeders is a reasonable consistency between molecular and phenotypic variation patterns. Such a consistency emerged in one pea study relative to a collection of 148 cultivars, breeding lines, and landraces genotyped with 121 protein- and PCR-based markers (Baranger et al., 2004), but failed to emerge in several studies carried out on different forage legumes, such as alfalfa (Crochemore et al., 1998), red clover (Greene et al., 2004; Pagnotta et al., 2011), and white clover (Kolliker et al., 2001). Pea molecular diversity studies, however, displayed variation patterns reflecting domestication (cultivated vs. wild types), phenological type, end-use (fodder, food or feed), and provenance from specific geographic regions, such as Eastern Africa, or Central and Eastern Asia (Baranger et al., 2004; Zong et al., 2009; Jing et al., 2010; Smýkal et al., 2011; Jing et al., 2012; Holdsworth et al., 2017; Siol et al., 2017; Hellwig et al., 2022; Pavan et al., 2022; Rispail et al., 2023).

A different approach to enhance the exploitation of large germplasm collections is the genotyping of the entire collection and the development of genomic prediction (GP) models trained on a subset of accessions to predict trait values of other accessions in germplasm collections and possibly in breeding populations. Prediction models proved valuable for pea breeding values of recombinant inbred lines for key polygenic traits such as crop yield in moisture-favorable (Annicchiarico et al., 2019b) and drought-prone environments (Annicchiarico et al., 2020), and protein content (Crosta et al., 2022) in pioneer studies. Other studies of yield prediction for grain legume germplasm accessions were encouraging, revealing predictive ability values not lower than 0.45 in soybean (Jarquín et al., 2016) and 0.40 in white lupin (Annicchiarico et al., 2019a) for the challenging scenario of cross-environment predictions (where model construction and validation are performed on data from distinct environments). Predictive ability values for large pea diversity panels including wild-relative genotypes, landraces and modern cultivars were reportedly moderate for grain yield and number of seeds per pod (Al Bari et al., 2021), high for number of seeds per plant and individual seed weight (>0.70) (Tayeh et al. (2015), and high (0.60-0.78) for onset of flowering (Tayeh et al., 2015; Al Bari et al., 2021). Molecular marker-based prediction of qualitative traits may also be useful in some cases, e.g., flower color, which is reportedly associated with seed tannin content, a trait potentially affecting grain protein digestibility in monogastric animals (Grosjean et al., 1998). The application of a genomic selection model constructed for a genetic base for predictions in another genetic base implies a penalty whose extent requires investigation. The predictive ability loss tended to be in the range of 40-50% for pea grain yield, protein content and other traits for inter-population predictions across RIL populations having one parent in common (Annicchiarico et al., 2019b; Annicchiarico et al., 2020; Crosta et al., 2022), while being unknown for other types of genetic bases.

The ever-increasing number of crop varieties, which approaches 3,400 cultivars in Europe and 6,000 globally for pea (Rubiales et al., 2021), complicates the assessment of the distinctness requirement according to morphological traits that is prescribed for the registration of new varieties according to UPOV (International Union for the Protection of New Varieties of Plants) regulations. Molecular-marker based distinctness has been advocated as a potentially quicker, more sensitive and lower-cost criterion to distinguish plant varieties compared with morphological trait-based distinctness used for verification of DUS (Distinctness, Uniformity and Stability) requirements for variety registration (Achard et al., 2020; Gilliland et al., 2020; Jamali et al., 2020). Molecular marker-based distinctness has been proposed as a complement or a substitute for the ordinary distinctness assessment in the presence of a reliable relationship with morphological trait-based diversity (Jones et al., 2013), a condition pending verification for pea. An ideal method of marker-based distinctness may rely on markers associated with morphological traits currently used for variety discrimination (UPOV, 2019).

The present study is based on phenotypic data collected by Annicchiarico et al. (2017) for a pea world germplasm collection including landraces, old cultivars and modern cultivars and GBS data for the same material reported in Pavan et al. (2022). Its objectives are: (a) to perform a genome-wide association study (GWAS) for seed protein content, grain yield under severe terminal drought, and other traits of possible interest for pea breeding or variety distinction; (b) to test the ability of GP models for protein content and other quantitative traits developed on the current germplasm panel to predict these traits in germplasm accessions and in breeding material as represented by an independent and much narrower genetic base including three Recombinant Inbred Line (RIL) populations evaluated in other Italian environments in earlier studies (Annicchiarico et al., 2019b; Crosta et al., 2022); (c) to investigate the consistency between molecular marker-based and phenotypic diversity patterns; (d) to verify the correspondence in terms of genomic position between genes that have already been cloned for qualitative traits and genomic regions highlighted as significantly associated to these traits by GWAS, and to detect yet unidentified genomic regions and alleles controlling qualitative and quantitative trait variation.

## 2 Materials and methods

### 2.1 Plant material and phenotyping

The study was based on 220 cultivated pea (*P. sativum* subsp. *sativum*) landraces and old cultivars belonging to 19 regional germplasm pools and 11 modern cultivars bred in France (Attika, Cartuce, Dove, Enduro, Genial, Isard, Messire, Spirale), Spain (Cigarron, Viriato) or Germany (Santana), evaluated by (Annicchiarico et al. (2017); **Supplementary Table 1**). This collection was set up by pooling selected accessions that were provided by IPK (Gatersleben), INRAE UMRLEG (Dijon), John Innes Centre (Norwich), CNR-IGV (Bari) and ICARDA’s gene bank. These institutions were asked to provide accessions which, according to the available knowledge, were able to maximize the genetic diversity within each country gene pool that was addressed by our request. A previous study (Pavan et al., 2022) confirmed the wide genetic variation and the absence of duplicates among the accessions represented in this collection.

This material was evaluated by Annicchiarico et al. (2017) in Lodi, Northern Italy (45°19’N, 9°03’E), in a spring-sown rain-fed field experiment designed as a randomized complete block with two replications. This experiment was characterized by substantial terminal drought associated with a rainfall amount of 178 mm over the crop cycle. The following traits were recorded on a plot basis: (i) dry grain yield (ii) dry aerial biomass, from which straw yield was derived by subtracting grain yield; (iii) onset of flowering (as days from January 1 to when 50% of the plants had the first open flower); (iv) individual seed weight; (v) color of the standard and of the rest of the flower (keel and wings), (vi) seed protein content, according to the Near Infrared Spectroscopy method as described in Annicchiarico et al. (2017); (vii) seed coat and hilum pigmentation, seed coat marbling and spotting, and cotyledon color and wrinkling, which were determined on the seed produced by the single plants employed for genotyping. Other experiment details can be found in Annicchiarico et al. (2017). Heterogeneity emerged occasionally for some morphological trait within landrace populations and even within progenies of individual plants, leading to exclusion of the accession from analyses for the relevant trait. In addition, we recorded anthocyanin pigmentation at stipule insertion on 160 accessions (158 landraces and two modern cultivars) in an unreplicated seed multiplication experiment performed during 2009 in Lodi.

A validation set for GP models developed for quantitative traits was represented by three RIL populations issued by connected crosses between three parent cultivars (Attika and Isard, of European origin; Kaspa, bred in Australia) that featured high and stable grain yield across Italian environments in earlier variety testing. This set included 306 lines that were evaluated by Annicchiarico et al. (2019b) and Crosta et al. (2022) for grain yield, grain protein content, onset of flowering and individual seed weight across three environments of Northern or Central Italy, and straw yield across two of these environments. Details about the experimental settings are given in these reports. These environments differed from the evaluation environment of the germplasm collection in various respects: they were autumn-sown, which implied substantial winter low temperature stress (particularly in one environment), more moisture-favorable (with at least 500 mm rainfall over the crop cycle), and managed organically.

### 2.2 Trait interrelationships

A chi-square test of independence (Rayner et al., 2011) was performed for all pairwise combinations of qualitative traits, which were expressed in a binary form, to investigate the occurrence of trait covariation. The phi coefficient (Guilford, 1941) was computed for each trait combination, providing a measure of the intensity and direction of association of the two variables. Other statistical analyses relative to variation and covariation of quantitative traits were reported in Annicchiarico et al. (2017).

### 2.3 DNA isolation, GBS library construction, and sequencing

For DNA extraction, one plant per accession was selected that represented the morphological characteristics of the entire accession based on visual observations. Information on DNA isolation and GBS-based genotyping can be found in Pavan et al. (2022) for the 231 accessions of the germplasm collection, and in Annicchiarico et al. (2019b) for lines belonging to the three RIL populations. The GBS analysis was outsourced to the Elshire Group by adopting Elshire et al.’s (2011) protocol with modifications, that is, using the *ApeK*I restriction enzyme and KAPA Taq polymerase.

The raw reads of accessions from the germplasm collection were pre-processed by Trimmomatic Version 0.39 (Bolger et al., 2014), aligned against pea reference genome v1a (Kreplak et al., 2019) by Burrows-Wheeler Aligner (Li and Durbin, 2009), and subjected to quality control and SNP calling within the dDocent pipeline (Puritz et al., 2014). Biallelic SNPs were selected and filtered for minor allele frequency (MAF) > 5%, missing rate < 20%, and heterozygosity rate < 30%, while accessions were filtered for missing rate < 25%.

The raw data of genotypes from the RIL populations were demultiplexed by axe demultiplexer (Murray and Borevitz, 2018), while pre-processing, alignment on reference genome version 1a (Kreplak et al., 2019) and SNP calling were performed by using the dDocent pipeline (Puritz et al., 2014). The final genotype matrix, in the form of a vcf file, was filtered for quality using the vcftool software (Danecek et al., 2011) with parameters –minQ 30, –max-non-ref-af 1, and –non-ref-af 0.001. RIL genotype data were merged with molecular data from the germplasm collection. Filtering of polymorphic SNPs was performed according to MAF > 5%, missing rate < 20%, and heterozygosity rate < 30%, while accessions were filtered for missing rate < 25%. Missing data were estimated by k nearest neighbour imputation method (Andridge and Little, 2010).

### 2.4 Analysis of phenotypic and genetic diversity patterns

Non-metric multi-dimensional scaling (NMDS; Kruskal, 1964) was applied to both phenotypic and molecular data of the germplasm collection, to provide a concise representation of accession diversity patterns and their consistency with geographic provenance for these information layers. NMDS was adopted in place of classical MDS, since the genetic dissimilarity coefficient that appeared more suitable for our genotype data, namely, Rogers’ distance (Rogers, 1972), is non-Euclidean (Gower and Legendre, 1986), whereas the Euclidean property represents a key assumption of classical MDS (Gower, 1985). Genotype data used for Rogers’ distance computation were pruned for linkage disequilibrium (LD) by snp.pruning() function from R package ASRgenomics, as suggested to avoid the strong influence of SNP clusters when estimating genetic relatedness (Laurie et al., 2010). A maximum *r*^2^ threshold of 0.2, a window size of 50 SNPs, and an overlap of 5 SNPs between consecutive windows were employed on the dataset formed by SNPs of known genomic position, generating a set of 11,072 SNPs. Dissimilarity for both qualitative and quantitative phenotypic traits, except for anthocyanin pigmentation at stipule insertion (which was eliminated from this analysis due to many accessions having missing data), was estimated by Gower’s distance (Gower, 1985). We investigated the correlation between genetic and phenotypic dissimilarity matrices by Mantel’s test (Mantel, 1967) using mantel() function from R package vegan (Dixon, 2003). The correlation between molecular and phenotypic diversity matrices was assessed with respect to all the SNPs on the one hand, and only the SNPs selected by the GWAS (including the significant SNPs for quantitative traits and the most significant SNP identified for each association peak of qualitative traits) on the other hand. This way, we verified the occurrence of a sharp rise of the correlation for a scenario of major interest for the molecular marker-based distinctness of variety germplasm in novel DUS procedures.

### 2.5 Analysis of population genetic structure

An analysis of population genetic structure was performed by the snmf() and Q() functions from the R package LEA (Frichot and François, 2015), which relies on different algorithms compared to STRUCTURE (Pritchard et al., 2000) but gives similar outputs and is considered more accurate for self-pollinating species (Frichot et al., 2014). Genotype data pruned for excess of LD as described in section 2.5 were employed. The optimal number of genetic clusters was visually selected based on the plot of the cross-entropy parameter, which was estimated by cross-validation (Alexander and Lange, 2011; Frichot et al., 2014). Genotypes were assigned to a cluster when featuring a minimum membership coefficient of 60%, otherwise they were classified as admixed.

### 2.6 Genome-wide association study and linkage disequilibrium decay

Population structure information to be included in the GWAS model was obtained by a Discriminant Analysis of Principal Components (DAPC; Yendle and MacFie, 1989) performed on genotype data pruned for excess of LD, as described in section 2.5. The k-means clustering algorithm was run iteratively for increasing values of K (i.e., numbers of genotype groups) from 1 to 30, to identify its optimal value according to differences between successive values of the Bayesian information criterion. The analysis was performed on the output of an ordinary principal component analysis to benefit from its dimensionality reduction but keeping all the components to avoid information loss. We performed the final DAPC by using the optimal K value. The number of principal components (PCs) to be retained for DAPC, and that of discriminant functions to be used as covariates in GWAS models, were determined by visual inspection of plots of PC cumulative variance and discriminant function eigenvalues, respectively. Based on this operation, 150 PCs were considered for DAPC and 8 discriminant functions were employed as GWAS covariates. The whole procedure was implemented by using the functions find.clusters() and dapc() from R package adegenet (Jombart and Ahmed, 2011).

LD was estimated as *r*^2^ value for pairwise combinations of SNPs within a 100 kb window by LD.decay() function from R package sommer (Covarrubias-Pazaran, 2016). The *r*^2^ values were plotted against physical distance and fitted by a polynomial curve as described in Marroni et al. (2011). The 90th percentile of the *r*^2^ distribution for pairwise combinations of SNPs located on different chromosomes was estimated by setting argument unlinked to TRUE in LD.decay() function, to assess the most meaningful LD decay threshold for candidate gene research in our dataset.

A GWAS was performed on 41,114 polymorphic SNPs according to (i) the Blink model (Huang et al., 2019) in R package GAPIT3 (Wang and Zhang, 2021) for quantitative traits, and (ii) a mixed logistic regression model by association.test.logistic() function from R package milorGWAS (Milet et al., 2020) for qualitative traits, which were unfitted to linear regression models due to their binary nature (Chen et al., 2016). The GWAS model for qualitative traits included the kinship matrix estimated by GRM() function from R package gaston as a covariate beside the DAPC components. To get an unbiased visual representation of type I errors (Chen et al., 2016), stratified quantile-quantile (QQ) plots were generated for qualitative traits by SNP.category() and qqplot.pvalues() functions from R package milorGWAS. These functions rely on the classification of SNPs in three categories depending on the ratio of expected variances in different population strata (Milet et al., 2020), which in our case were defined by DAPC cluster membership. Visual examination of QQ plots for both qualitative (**Supplementary Figure 1**) and quantitative traits (**Supplementary Figure 2**) highlighted an appropriate compensation of population structure by GWAS model covariates, except for hilum pigmentation, seed coat marbling, and cotyledon wrinkling, which exhibited either some over- or under-compensation depending on the relevant SNP category. A Bonferroni threshold of 5% was employed to select significant SNPs for all traits. The exact genomic position of previously cloned genes controlling qualitative traits was determined by BLAST alignment of either DNA or protein sequences and selection of genomic sequences showing 100% homology.

### 2.7 Genomic regression models

Genomic predictions were investigated for all quantitative traits (grain yield, straw yield, protein content, onset of flowering, and individual seed weight) by using three statistical models, namely, Ridge regression BLUP (rrBLUP; Meuwissen et al., 2001), BayesC (Habier et al., 2011), and Bayesian Lasso (Park and Casella, 2008) within the R package GROAN (Nazzicari and Biscarini, 2017). The rrBLUP model assumes that marker effects have a common variance, which makes it more suitable for traits controlled by a large number of quantitative trait loci (QTL) with a small effect, whereas Bayesian models assume relatively few markers with large effects, therefore allowing for different marker effects and variances (Wang et al., 2018). Predictions were assessed for two scenarios. The former, which relied on 41,114 SNPs for GP model construction, consisted in a ten-fold non-stratified cross-validation performed on germplasm collection data with 50 repetitions for rrBLUP and 10 for Bayesian models. Predictive ability results (*r*_Ab_, computed as Pearson’s correlation between the observed phenotypic values and those predicted by the model) were obtained by averaging repetition values. The latter, more challenging scenario envisaged an inter-population, inter-environment validation of GP models, which were constructed from data of the germplasm collection and were validated for predictive ability on data of each RIL population and on the pooled data of the populations (the latter representing a more diversified breeding line panel relative to the individual populations). Only 4,929 SNPs shared by the germplasm collection and the RIL material were available for GP model validation. Recombinant inbred line data were previously averaged across validation environments from Northern and Central Italy, which belong to the same target region (meaning that the within-site year-to-year climatic variation affects the genotype yield responses more than the geographic distance between sites: Annicchiarico and Iannucci, 2008).

## 3 Results

### 3.1 Phenotypic variation and trait interrelationships

Phenotypic variation within geographic pools resulted significant at *p* < 0.01 for all quantitative traits in Annicchiarico et al. (2017), to which we refer for further details about the variability within and between pools. The high impact of terminal drought was confirmed by the low mean grain yield (about 1.1 t/ha) displayed by the material. On average, modern cultivars, compared with the traditional germplasm, displayed lower grain and straw yield in spite of an earlier flowering, similar protein content, and higher individual seed weight (**Table 1**). However, the range of phenotypic variation was remarkably larger for the landrace and old cultivar group compared with the improved variety group for all the quantitative traits (**Table 1**). Broad-sense heritability values were fairly modest for grain yield, moderately high for straw yield and protein content, and very high for onset of flowering and individual seed weight (**Table 1**). The modern cultivars were semi-leafless and mostly displayed a white flower, in contrast with the traditional germplasm that was leafy and with a higher proportion of purple-flowered genotypes (**Table 2**). Yellow cotyledon color, smooth cotyledon and white hilum were the dominant phenotypes in both landrace and old cultivar material and in modern germplasm (in which no seed showed wrinkling or a pigmented hilum) (**Table 2**). Seed coat pigmentation, marbling, and spotting, and anthocyanin pigmentation of stipules were relatively frequent phenotypes in the traditional germplasm, while being completely absent or rare in modern cultivars (**Table 2**).

**Table 1:** Mean, range of variation, and broad-sense heritability (*H^2^*) estimated on a genotype mean basis, for five quantitative traits measured on a world pea germplasm collection of 220 landraces from 19 regional pools and 11 modern cultivars.

Trait	Landraces		Improved varieties		*H^2^*
	Mean	Range	Mean	Range	
Grain yield (t/ha)	1.11	0.16 - 3.30	0.85	0.31 - 2.03	0.47
Straw yield (t/ha)	2.01	0.47 - 5.73	1.62	0.88 - 2.89	0.70
Protein content (g/100 g)	22.8	17.5 – 27.8	22.8	20.4 - 23.2	0.68
Onset of flowering (dd from Jan. 1)	133	113 - 154	131	127 - 136	0.87
Individual seed weight (mg)	138	52 - 277	157	107 - 211	0.91

**Table 2:** Qualitative traits of a world pea germplasm collection of 220 landraces from 19 regional pools and 11 modern cultivars.

Trait	Landraces^a^	Improved varieties
Leaf Type		
Leafy	220	0
Semi-leafless	0	11
Standard pigmentation		
Purple	119	1
White	101	10
Wing and keel pigmentation		
Purple	137	1
White	83	10
Cotyledon color		
Green	22	1
Yellow	175	10
Seed coat pigmentation		
Present	117	0
Absent	90	11
Hilum pigmentation		
Present	45	0
Absent	162	11
Cotyledon wrinkling		
Absent	193	11
Present	16	0
Stipule pigmentation^b^		
Present	94	0
Absent	64	2
Seed coat marbling		
Present	34	0
Absent	175	11
Seed coat spotting		
Present	42	0
Absent	164	11

a Accessions displaying trait heterogeneity were excluded from analyses for the trait.

b Observed on a subset of 160 accessions.

Chi-square tests of independence highlighted several significant associations between qualitative traits. High positive associations were observed among all the traits related to pigmentation of vegetative or reproductive organs, namely, stipule pigmentation, purple flower standard, purple flower keel and wings, and pigmented seed coat (phi coefficient ≥ 0.72, *p* < 0.001) (**Table 3**). High phenotypic correlation (*r* ≥ 0.70) among quantitative traits was only observed between grain and straw yield, while no correlation emerged between grain yield and protein content (*r* = 0.01), as reported more in detail in Annicchiarico et al. (2017).

**Table 3:** Phi coefficient of association for pairwise combinations of ten qualitative traits measured on a world pea germplasm collection of 220 landraces from 19 regional pools and 11 modern cultivars.

Trait	Stipule pigmentation	Pigmented seed coat	Marbled seed coat	Spotted seed coat	Pigmented hilum	Wrinkled cotyledon	Yellow cotyledon	Purple standard	Purple keel and wings	Presence of leaves
Stipule pigmentation		0.78***	0.16 NS	0.39***	0.35***	0.02 NS	0.18*	0.73***	0.72***	0.07 NS
Pigmented seed coat			0.40***	0.46***	0.47***	-0.17 NS	0.25 NS	0.86***	0.86***	0.25 NS
Marbled seed coat				-0.18**	0.40***	-0.12 NS	0.07 NS	0.37**	0.36***	0.10 NS
Spotted seed coat					0.32**	-0.14 NS	0.14 NS	0.43***	0.39***	0.11 NS
Pigmented hilum						-0.14 NS	0.18 NS	0.38**	0.43***	0.12 NS
Wrinkled cotyledon							-0.39 NS	-0.18 NS	-0.19 NS	0.06 NS
Yellow cotyledon								0.23 NS	0.28*	-0.01 NS
Purple standard									0.85***	0.19 NS
Purple keel and wings										0.23 NS
Presence of leaves										

*p < 0.05; **p < 0.01; ***p < 0.001; NS, not significant (p > 0.05).

### 3.2 Analysis of phenotypic and genetic diversity patterns

**Figure 1:**
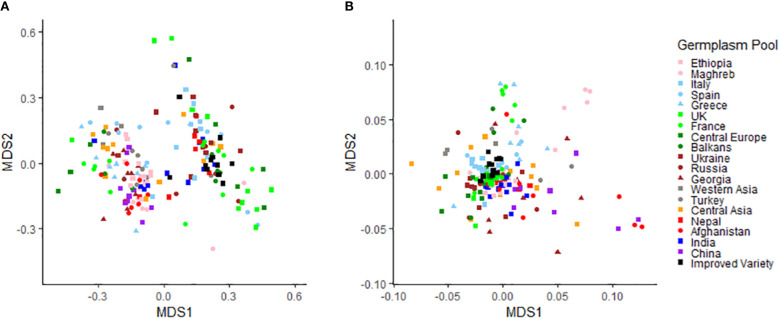
Results of non-metric multi-dimensional scaling analyses based on **(A)** phenotypic data for eight qualitative and five quantitative traits and **(B)** molecular data of 11,072 SNPs for a worldwide pea germplasm collection of 220 landraces from 19 regional pools and 11 modern cultivars.

The value of the NMDS stress function, representing a measure of rank-order disagreement between observed and fitted distances, was equal to 0.15 and 0.21 for phenotypic and molecular data, respectively. The higher and somewhat sub-optimal stress value found for molecular data could be related to their much greater number of original variables (SNPs) compared with phenotypic data (implying greater information loss by the two-dimensional representation). The NMDS performed on phenotypic data revealed no distinct pattern of variation related to the geographic origin of the material, albeit with a trend of European landraces and modern cultivars towards positive values along the first axis (**Figure 1A**). The morphological diversity of the modern cultivars bred in France, Spain or Germany was distinctly narrower than that of the traditional germplasm from these countries (**Figure 1A**). Although the diversity patterns based on molecular data were poorly related to the geographic origin of the material, various accessions for China, Afghanistan and Maghreb stood out for being genetically distinct from the rest of the germplasm (**Figure 1B**). The modern germplasm revealed particularly narrow genetic diversity on the ground of molecular data (**Figure 1B**). Mantel’s test highlighted quite a modest albeit significant correlation (*r* = 0.12, *p* < 0.01) between accession dissimilarity matrices based on phenotypic and overall molecular information. The correlation increased remarkably (*r* = 0.45) when the analysis referred only to the molecular information accounted for by significant SNPs detected by GWAS analyses reported afterwards.

### 3.3 Analysis of population genetic structure

**Figure 2:**
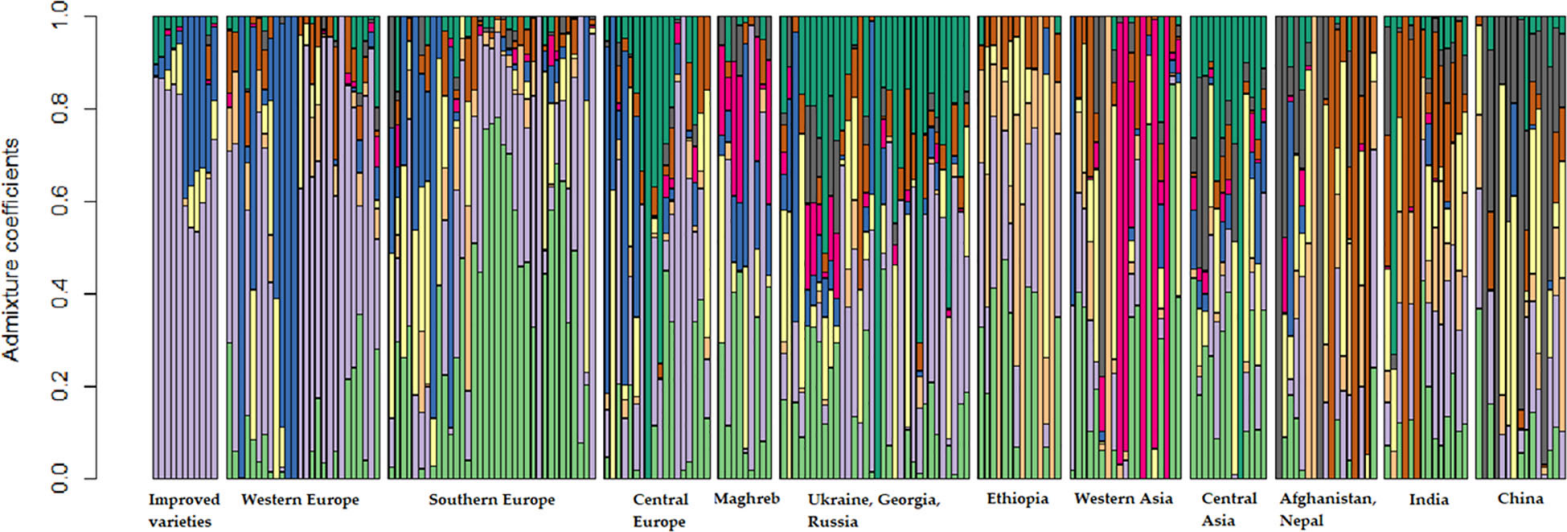
Results of a population structure analysis with K = 9 performed on the molecular data of 11,072 SNPs for a worldwide pea germplasm collection of 220 landraces and 11 modern cultivars. Each color represents a specific number of genotype groups (K). Results are displayed for the improved variety group and for 11 landrace regional pools that partly merge the initial 19 pools, ordered from West to East.

The optimal number of genetic clusters was equal to 9 (**Supplementary Figure 3**). This classification analysis confirmed the results of the NMDS with respect to the quite restricted molecular diversity of the improved variety pool, of which the large majority of genotypes was assigned to the same cluster (violet cluster in **Figure 2**). The same cluster accommodated most of the landrace and old cultivar accessions from Western Europe, the region in which most of the modern germplasm originated (**Figure 2**). Southern Europe material included some admixed genotypes, but also showed some genetic specificity as revealed by many accessions attributable to a single cluster (the light green one in **Figure 2**). Asian pools were highly differentiated both from each other and from material from other continents, with some clusters that showed up only in specific geographic pools, as in the cases of Western Asia (fuchsia color in **Figure 2**), South-Central Asia as represented by Afghanistan, Nepal, and India (brown color in **Figure 2**), and China (grey color in **Figure 2**). Differentiation emerged also for a subset of the Ethiopian accessions (orange color in **Figure 2**). Most of the remaining geographic pools were largely characterized by admixed genotypes (**Figure 2**).

### 3.4 Genome-wide association study and linkage disequilibrium decay

On average, LD reached half of its 90^th^ percentile (*r*^2^ = 0.38) at 217 bp, with single chromosome values ranging from 146 bp for chromosome 2 to 326 bp for chromosome 4 (**Supplementary Figure 4**). The 90th percentile of the *r*^2^ distribution for pairwise combinations of SNPs located on different chromosomes resulted equal to 0.05 and was reached at 10,140 bp on average (**Supplementary Figure 4**). The mean distance at which *r*^2^ dropped to 0.05 on a specific chromosome was scanned in both directions from each significant SNP on that chromosome to look for candidate genes.

**Figure 3:**
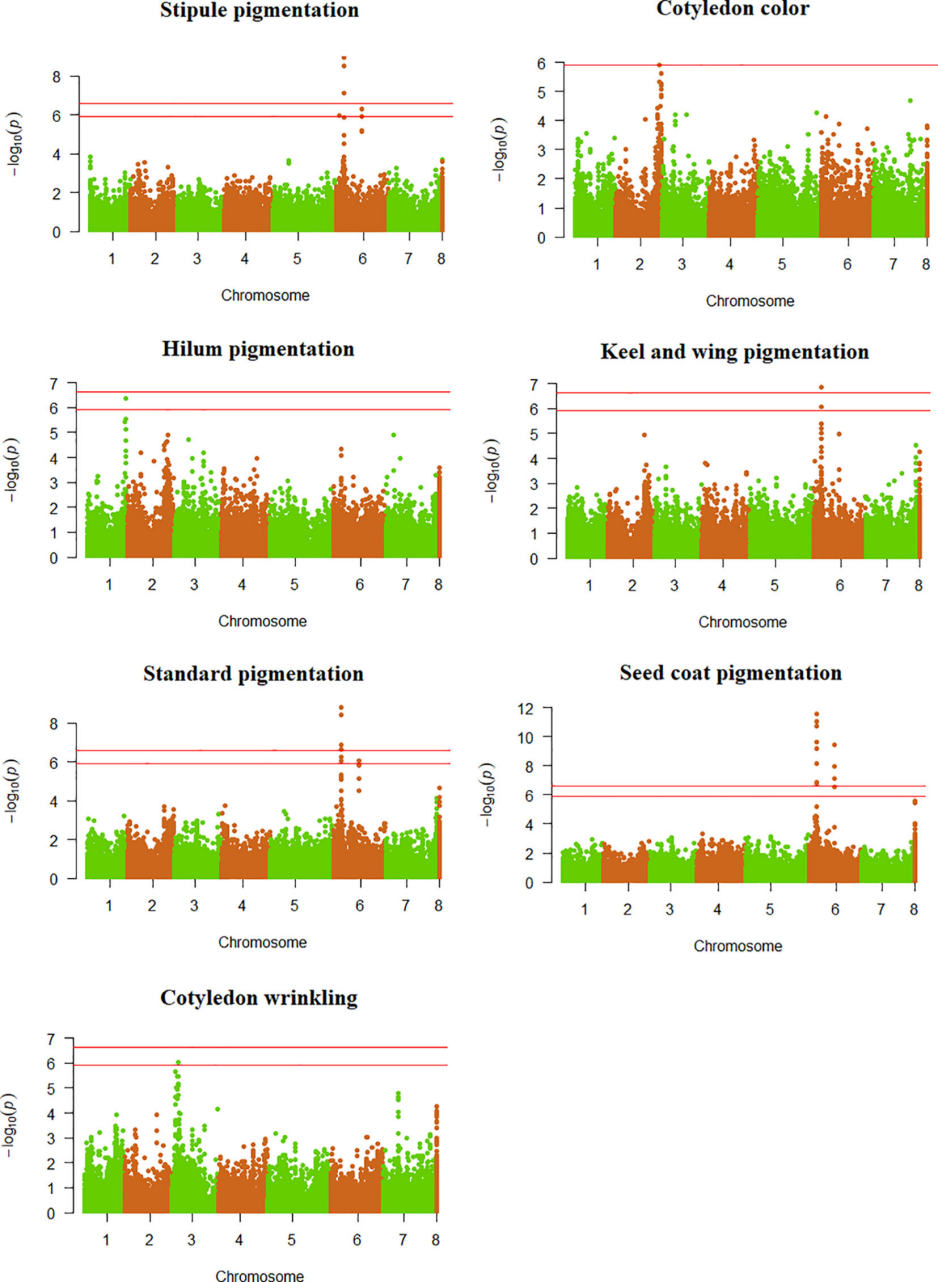
Manhattan plots showing the association scores of 41,114 SNPs with seven qualitative traits along pea chromosomes for a GWAS based on a worldwide germplasm collection of 220 landraces from 19 regional pools and 11 modern cultivars. The red lines represent Bonferroni threshold at 5% and 1%.

The DAPC was performed by adopting K = 16 as the optimal group number. Various accessions from Western Asia stood out as quite different from other germplasm pools in the space of the first two axes of the DAPC (**Supplementary Figure 5**). The list of significant SNPs detected for qualitative and quantitative traits is provided in **Supplementary Table 2** along with additional information about their MAF and estimated effect, while a list of the relative candidate genes is reported in **Supplementary Table 3**. Significant SNPs were found in the same genomic regions of previously cloned genes for flower standard, keel and wing pigmentation (Hellens et al., 2010), hilum pigmentation (Balarynová et al., 2022), and cotyledon wrinkling (Bhattacharyya et al., 1990) (**Figure 3**; **Supplementary Table 3**). Although the significant SNPs appeared quite close to the cloned sequences for all traits, chromosome estimates of LD decay prevented us from inferring a clear linkage. Therefore, we estimated the LD between the significant SNPs and the first SNP on the opposite side of the cloned locus. For all traits, *r*^2^ values higher than the empirical threshold of 0.05 were found between one or more significant SNPs and one or more SNPs on the opposite side of the cloned locus, supporting the potential correspondence between the observed association peaks and the previously cloned genes (**Supplementary Table 4**).

The GWAS confirmed the largely pleiotropic control of traits related to pigmentation of vegetative or reproductive organs that was suggested by estimates of phi coefficients. Seven significant SNPs were found for the color of the flower standard (**Figure 3**). Six of them formed a single peak on chromosome 6 (ranging between 67.6 and 68.4 Mb) close to the A locus that encodes a transcription factor likely involved in the regulation of the anthocyanin pathway (Hellens et al., 2010), while the remaining SNP determined a second peak on the same chromosome at 235.6 Mb (**Supplementary Table 2**). The two SNPs featuring the highest significance level for standard color, mapping in the peak potentially linked to the A locus, corresponded to the only two significant SNPs found for keel and wing color (**Figure 3**; **Supplementary Table 2**). For anthocyanin pigmentation of stipules, we identified five significant SNPs on chromosome 6, of which three mapped in the A locus region, one in the 235.6 Mb region, and the last one upstream of the A locus region at 27.4 Mb (**Figure 3**; **Supplementary Table 2**). Two significance peaks emerged for seed coat pigmentation on chromosome 6, the first containing nine SNPs located in the A locus region, and the second including four SNPs mapping in the 235.6-235.8 Mb region (**Figure 3**; **Supplementary Table 2**).

One significant SNP each emerged for cotyledon wrinkling and hilum pigmentation. The former located on chromosome 3 close to the R_a_ locus, which encodes a starch branching enzyme (Bhattacharyya et al., 1990) (**Figure 3**; **Supplementary Tables 2** and **3**); the latter mapped on chromosome 1 near to the locus Pl, which encodes a polyphenol oxidase enzyme (Balarynová et al., 2022) (**Figure 3**; **Supplementary Tables 2** and **3**). No significant association was found for seed coat spotting and marbling, and cotyledon color (**Supplementary Figure 6**).

**Figure 4:**
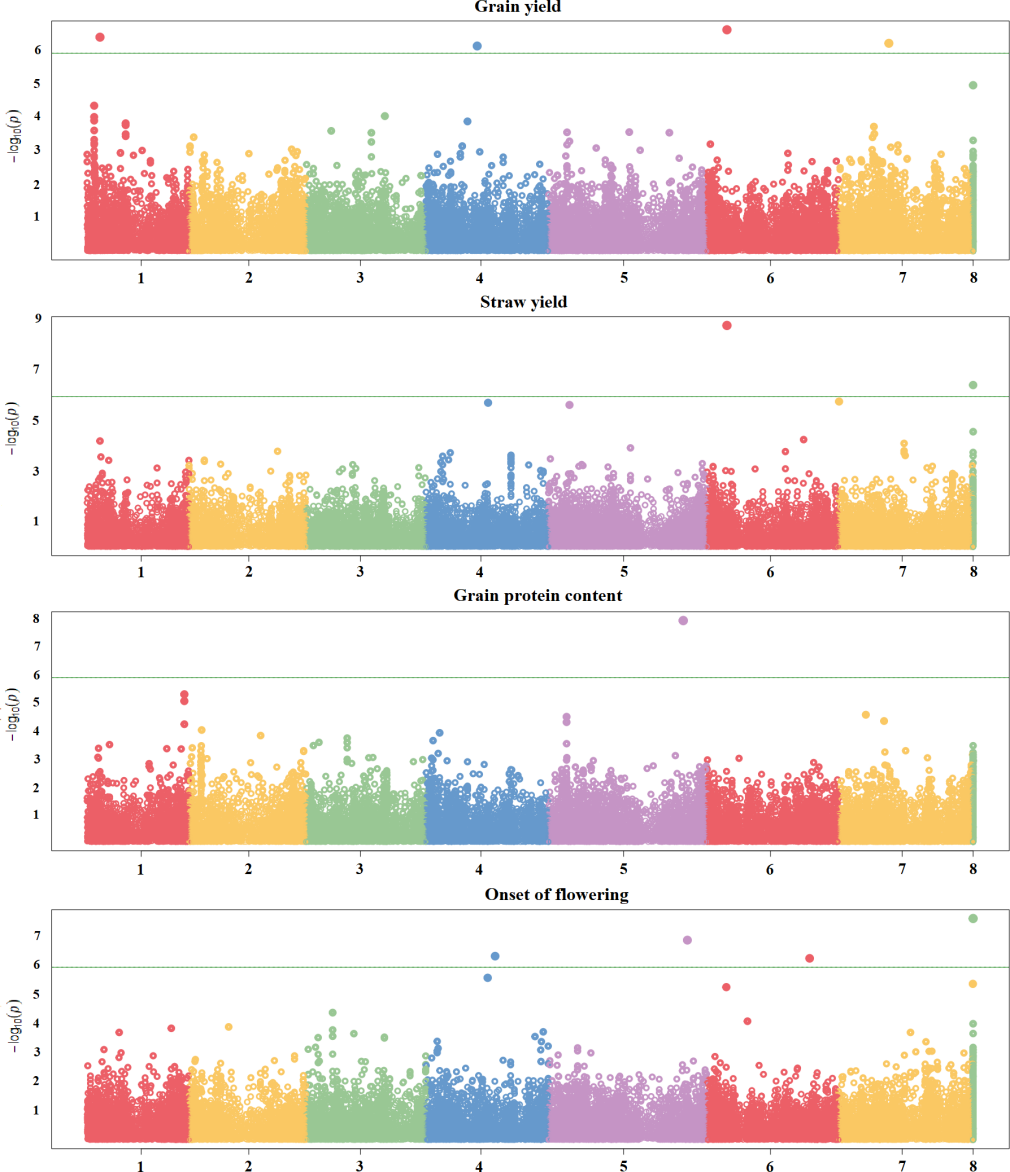
Manhattan plots showing the association scores of 41,114 SNPs with four quantitative traits along pea chromosomes for a GWAS based on a worldwide germplasm collection of 220 landraces from 19 regional pools and 11 modern cultivars. The green line represents Bonferroni threshold at 5%.

Three quantitative traits, i.e., grain yield and straw yield under severe terminal drought, and onset of flowering, displayed a few significant associations. Four significant SNPs mapping on chromosomes 1, 4, 6 and 7 were found for grain yield, whereas two significant SNPs were identified for straw yield on chromosomes 6 and scaffolds (**Figure 4**; **Supplementary Table 2**). Interestingly, the significant SNPs found for grain yield and straw yield on chromosome 6 were coincident (**Figure 4**; **Supplementary Table 2**). Four significant SNPs were identified for onset of flowering, of which three mapped on chromosomes 4, 5 and 6, and one was located on scaffolds (**Figure 4**; **Supplementary Table 2**). In contrast, just one significant SNP placed on chromosome 5 emerged for grain protein content (**Figure 4**; **Supplementary Table 2**), and no significant association was detected for individual seed weight (**Supplementary Figure 6**), although some SNPs approaching significance emerged for both of these traits.

### 3.5 Genomic regression models

The GP models trained and validated on the germplasm collection displayed moderate to high predictive ability (*r*_Ab_) for all traits, mostly with slight differences between statistical models (**Table 4**). In particular, the predictive ability could be considered as moderate for a genetically complex trait such as grain yield (*r*_Ab_ = 0.435 for the best model), moderately high for grain protein content, straw yield and onset of flowering (*r*_Ab_ in the range of 0.55-0.62), and high for individual seed weight (*r*_Ab_ = 0.764; **Table 4**).

**Table 4:** Predictive ability based on a ten-fold intra-population intra-environment cross-validation for five quantitative traits, using three genomic prediction models and 41,114 polymorphic SNPs of a world pea germplasm collection of 220 landraces from 19 regional pools and 11 modern cultivars.

Trait	Ridge regression BLUP	Bayesian Lasso	Bayesian C
Grain yield	0.435	0.431	0.426
Straw yield	0.578	0.576	0.575
Protein content	0.549	0.540	0.539
Onset of flowering	0.608	0.618	0.613
Individual seed	0.763	0.737	0.764

As expected, the challenging scenario of predictions for the much narrower genetic base represented by the three RIL populations based on the model constructed from data of the germplasm collection, which could rely on 4,929 polymorphic SNPs shared by the two genetic bases, produced a substantial decrease of predictive ability values. However, the decrease varied depending on the traits and the RIL population. With respect to predictions for the whole set of inbred lines, the predictive ability reduction estimated from comparison of top-predicting models approached 40% for individual seed weight (0.470 vs. 0.764), 50% for protein content (0.281 vs. 0.549) and straw yield (0.313 vs. 0.578), and 60% for onset of flowering (0.261 vs. 0.618). The predictive ability close to zero observed for grain yield (**Table 4**) was probably influenced by the contrasting evaluation environments of the two genetic bases (as reflected by much greater yielding ability values displayed by RIL material compared with germplasm accessions: **Tables 1** and **5**). High predictive ability values were observed for specific trait-RIL population combinations, as in the case of grain or straw yield of RIL material originated from the European cultivar Isard and the Australian cultivar Kaspa, which exhibited nearly no loss of predictive ability compared with intra-population, intra-environment predictions (**Tables 4** and **5**). Predictions for the RIL originated from the two European cultivars (Attika and Isard) approached zero for grain yield and protein content but were valuable for seed weight (**Table 5**). The extent of within-RIL population phenotypic variation was similar for nearly all traits (**Table 5**), suggesting that other factors may account for the observed differences for within-RIL population predictive ability.

**Table 5:** Trait range values and predictive ability based on inter-population, inter-environment validation for five quantitative traits, using three genomic prediction models constructed from data of a world pea germplasm collection of 220 landraces from 19 regional pools and 11 modern cultivars and validated on data of 306 modern inbred lines belonging to three connected RIL populations.

Trait^a^	Validation material^b^	Range values	Predictive ability		
			Ridge regression BLUP	Bayesian Lasso	Bayesian C
Grain yield (t/ha)	RILs A×I	2.79 - 6.79	−0.236	-0.237	-0.246
Grain yield (t/ha)	RILs K×A	2.09 - 6.05	0.270	0.258	0.264
Grain yield (t/ha)	RILs K×I	3.08 - 7.60	0.446	0.439	0.443
Grain yield (t/ha)	All RILs	2.79 - 7.60	−0.025	-0.038	-0.022
Straw yield (t/ha)	RILs A×I	2.23 - 7.28	0.265	0.266	0.277
Straw yield (t/ha)	RILs K×A	1.91 - 7.02	0.232	0.207	0.216
Straw yield (t/ha)	RILs K×I	2.52 - 9.91	0.518	0.512	0.518
Straw yield (t/ha)	All RILs	1.91 - 9.91	0.313	0.295	0.302
Protein content (g/100 g)	RILs A×I	21. 7 - 25.8	-0.225	-0.240	-0.190
Protein content (g/100 g)	RILs K×A	22.0 - 26.6	0.028	0.024	-0.013
Protein content (g/100 g)	RILs K×I	22.5 - 26.4	0.184	0.185	0.157
Protein content (g/100 g)	All RILs	21.7 - 26.7	0.281	0.263	0.255
Onset of flowering (dd from Apr. 1)	RILs A×I	6 - 17	0.240	0.227	0.233
Onset of flowering (dd from Apr. 1)	RILs K×A	8 - 25	0.169	0.174	0.167
Onset of flowering (dd from Apr. 1)	RILs K×I	6 - 23	0.201	0.264	0.247
Onset of flowering (dd from Apr. 1)	All RILs	6 - 25	0.244	0.251	0.261
Individual seed weight (mg)	RILs A×I	0.158 - 0.242	0.539	0.542	0.537
Individual seed weight (mg)	RILs K×A	0.176 - 0.261	0.268	0.267	0.268
Individual seed weight (mg)	RILs K×I	0.149 - 0.239	0.055	0.066	0.056
Individual seed weight (mg)	All RILs	0.149 - 0.261	0.461	0.470	0.464

Analyses based on 4,929 polymorphic SNPs shared by the two germplasm sets.

a Evaluation in two (straw yield) or three (other traits) environments of Northern or Central Italy.

b RIL parent material identified by A for Attika, I for Isard and K for Kaspa. A and I, European origin; K, Australian origin.

## 4 Discussion

Our joint investigation of phenotypic and molecular diversity patterns and Mantel’s test results indicated the substantial inconsistency between phenotypic and molecular diversity, in contrast with earlier results for pea by Baranger et al. (2004) but in agreement with several studies on other legume species (Crochemore et al., 1998; Kolliker et al., 2001; Greene et al., 2004; Pagnotta et al., 2011). This finding would set a limit to our ability to define core collections solely on the ground of molecular information from random markers. The large increase in the consistency between phenotypic and molecular diversity indicated by Mantel’s test when the latter diversity was estimated from GWAS-selected markers encourages the definition of molecular marker-based criteria for variety distinctness strictly related to morphological diversity for DUS traits, aimed to complement or possibly substitute the current morphological trait-based criteria. Molecular marker-based criteria, especially if they could be based on relatively large marker numbers, may offer several potential advantages for DUS testing relative to those based on morphological traits, such as a faster and cheaper application, independence from testing conditions, and greater suitability for lawsuits (Gilliland et al., 2020). The genetic diversity relative to markers associated with agronomic and morphological traits may also be exploited for the selection of core collections able to maximize the genetic variation for traits that are relevant to breeders. This approach would be definitely valuable if it was based on markers linked to a more comprehensive set of traits than the current one, including, for example, the tolerance to several key abiotic and biotic stresses.

The results of NMDS and the analysis of population genetic structure indicated a modest correspondence between molecular diversity and geographic origin of the landraces and old cultivars. These analyses and the DAPC highlighted the noticeable level of genetic differentiation characterizing materials from Western Asia, which represents the primary domestication center for pea (Zohary and Hopf, 1973). The gradual change in predominant clusters observed in the analysis of population genetic structure along a West-East gradient (**Figure 2**), with most of the intermediate pools featuring a considerable proportion of admixed accessions, agrees with earlier studies by Jing et al. (2012) and Rispail et al. (2023) and with results by Pavan et al. (2022). The latter study suggested two major routes of pea introduction into cultivation starting from West Asia, one westward along the northern and southern shores of the Mediterranean region, and another eastward towards Central Asia. The relatively high molecular differentiation that we observed for traditional germplasm from Eastern Asia, especially China, and Afghanistan, agrees with this hypothesis and with earlier findings by Zong et al. (2009) and Smýkal et al. (2011). The moderate level of genetic distinction that we found for germplasm from Maghreb may derive from the edge position of this region along the westward expansion of the crop from the Fertile Crescent. Ethiopian germplasm, which is known to originate from a separate domestication event (Trněný et al., 2018; Weeden, 2018) and displayed marked genetic distinctness from other landrace germplasm in the studies by Hellwig et al. (2022) and Rispail et al. (2023), currently showed moderate differentiation, in agreement with the results of an earlier study based on pooled data from the USDA and the current collection (Pavan et al., 2022).

An additional result that emerged consistently from our NMDS and population structure analyses was the limited genetic diversity of the improved cultivars bred in Western Europe relative to that displayed by landraces and old cultivars. This finding, which agrees with results reported by Baranger et al. (2004), has considerable importance for breeding programs, indicating the large availability of untapped genetic variation for broadening the crop genetic diversity. This finding reinforces the practical interest of identifying genome-based tools that could ease the mining of germplasm collections for traits of primary importance for crop improvement.

The observed LD decay was much faster than that reported by Alemu et al. (2022) for a collection of 188 vining pea varieties and breeding lines provided by a single company (where *r^2^ = *0.2 was reached at 6,930,000 bp on average vs. 1,445 bp in our study), while being slower compared to what reported by Pavan et al. (2022) for a larger germplasm collection (where *r^2^ = *0.2 was reached at 30 bp on average). These results are in substantial accordance with expectations, considering that the first genetic base was likely much narrower, while the second one was more diversified, relative to the current one. However, these studies adopted different LD estimation methods compared with our study, and this may have some bearing on the results. The fast LD decay value featuring our data set would ensure an almost single gene resolution (helpful for the identification of candidate genes).

Despite the somewhat sub-optimal germplasm sample size, the GWAS was able to detect several significant associations for both qualitative and quantitative traits, which, in addition to their possible exploitation for breeding purposes, could help in the definition of marker sets for the assessment of variety distinctness or core collection set up. Significance peaks potentially associated to the A locus were identified for the anthocyanin pigmentation of standard, keel and wings, seed coat and stipules, in accordance with the reported role of this gene in the regulation of the anthocyanin pathway (Hellens et al., 2010). We identified additional significance peaks for all these traits, except for keel and wing pigmentation. Seed coat and stipule pigmentation shared the two peaks identified for standard pigmentation, with the latter displaying an additional peak located on the same chromosome (**Figure 3**; **Supplementary Table 2**). These findings suggest that the genetic control may rely on both constitutive and local regulation mechanisms, at least for some of the anthocyanin pigmentation traits.

Our results for hilum pigmentation and cotyledon wrinkling confirmed largely what reported in previous gene mapping studies. We failed to detect any significant association for cotyledon color and seed coat marbling. However, the most significant SNP for the former trait, located on chromosome 2, was extremely close to Bonferroni threshold at 5% (**Figure 3**) and to the cloned locus I (Sato et al., 2007). For the latter trait, the ten SNPs featuring the highest association score mapped on chromosome 5, in accordance with findings by Murfet (1973) (**Supplementary Figure 6**).

Burstin et al. (2007) and Klein et al. (2020) identified significant QTLs for grain protein content in the same genomic region of chromosome 5 in which we found the only significant SNP for this trait. For onset of flowering, significant loci were found in genomic regions close to our significant SNPs by Gali et al. (2019) on chromosome 4 and by Klein et al. (2014) on chromosome 5.

Several QTLs were detected for individual seed weight in various studies performed under moisture-favorable growing conditions (Irzykowska and Wolko, 2004; Burstin et al., 2007; Krajewski et al., 2012; Klein et al., 2014; Gali et al., 2018; Gali et al., 2019; Klein et al., 2020). The current lack of significant SNPs in the presence of large phenotypic variation and high genome-enabled predictive ability indicated a genetic control of seed weight based on many small-effect genes in this study. While the drought stress of our phenotyping environment may have decreased our ability to identify QTLs by flattening the genetic variation and reducing the effect of genes conferring a heavier seed, the definitely quantitative genetic architecture of this trait was confirmed by its good genomic predictive ability even for a different genetic base grown under moisture-favorable conditions (i.e., the three sets of RILs).

Significant loci for grain yield were detected in the same genomic regions of our significant SNPs by Gali et al (2018; 2019) on chromosome 1, by Irzykowska and Wolko (2004) and Burstin et al. (2007) on chromosome 4, and by Burstin et al. (2007) on chromosome 7. The consistency observed between our results and those from other studies is remarkable as the latter were obtained in much more favorable conditions in terms of water availability compared with ours, suggesting that the reported markers may be relevant across a wide range of environmental conditions. The significance of the SNP found on chromosome 6 for both grain and straw yield suggests that it may have an impact on source (i.e., radiation and/or water use efficiency) rather than sink (i.e., harvest index) mechanisms under the current growing conditions.

Our study confirmed a widespread polygenic control of quantitative traits (grain and straw yield, protein content, seed weight, onset of flowering), emphasizing the interest to develop GP models. The predictive ability values of GP models generated by intra-population, intra-environment cross-validations were comparable with those reported for pea germplasm collections. In particular, our values of 0.43 for grain yield and 0.62 for onset of flowering for top-predicting models are nearly identical to those reported for these traits in the USDA pea collection by Al Bari et al. (2021), while our value of 0.76 for individual seed weight is only slightly lower than that reported for a broad germplasm collection by Tayeh et al. (2015). The whole of these results, and the predictive ability value of 0.55 reported here for a key quality trait such as protein content, are quite encouraging for the identification of elite genetic resources in large germplasm collections by GP models.

Our application of GP models defined from data of a world germplasm collection to predict breeding line values was challenged by the much narrower genetic base of the target germplasm, the over eight-fold reduction of available SNP markers shared by the two genetic bases (4,929 vs. 41,114), and the large differences between evaluation environments in terms of sowing time (autumn vs. spring) and extent of drought stress (limited vs. severe). The lack of predictive ability of the GP model for grain yield that we found for the whole set of lines is not surprising in this context. It is noticeable, however, the moderately high predictive ability value (0.446) exhibited by the GP model for grain yield of the RILs issued by the cross of Kaspa × Isard, namely, two parents with contrasting geographic origin and large Nei’s genetic distance compared with that between the two European cultivars (Attika and Isard) (Annicchiarico et al., 2019a). A similar result was obtained for within-RIL population predictions for protein content. These findings suggests the opportunity of a prior assessment of the predictive ability of the generated models for specific breeding material based on a relatively small subset of lines.

The loss of predictive ability for the whole set of breeding lines observed for the other traits (around 40% for individual seed weight, 50% for protein content and straw yield, and 60% for onset of flowering) was lower than expected, when considering the circumstances and the fact that a comparable loss was observed for protein content, onset of flowering and seed weight for inter-population, inter-environment predictions relative to RIL populations that differed for one parent genotype and were evaluated in much more similar test environments (Annicchiarico et al., 2019b; Crosta et al., 2022). In general, these models kept some interest for trait prediction of modern breeding material in the absence of more germplasm-specific GP models, while showing greater predictive ability for specific germplasm sets (as represented by individual RIL populations) that ought to be verified preliminarily.

In conclusion, our study generated information on genomic areas involved in the control of several morphological and agronomic traits that could be used for mining useful genetic resources within large germplasm collections. Our results could also contribute to the definition of procedures for molecular marker-based discrimination of varieties proposed for registration and the setting up of core collections. In addition, we generated genomic prediction models that proved sufficiently accurate for identifying elite genetic resources with greater yielding ability and/or specific seed traits (protein content and seed size) and phenology, holding a possible interest also for genomic selection in breeding programs. On the whole, our results highlighted the usefulness of genotyping data for a cost-effective exploitation of genetic resources.

## Data availability statement

Germplasm collection data are available as raw FASTQ files deposited at the SRA database under the BioProject identification number PRJNA719084 (https://www.ncbi.nlm.nih.gov/bioproject/719084). RIL population data are available in Additional file 6, Archive S1 at https://bmcgenomics.biomedcentral.com/articles/10.1186/s12864-019-5920-x.

## Author contributions

MC: Writing – original draft, Data curation, Formal analysis, Investigation, Methodology, Visualization. MR: Data curation, Investigation, Writing – review & editing. NN: Data curation, Methodology, Software, Writing – review & editing. BF: Data curation, Investigation, Writing – review & editing. PA: Conceptualization, Data curation, Funding acquisition, Investigation, Methodology, Project administration, Supervision, Writing – original draft.
