# Supplementary material for: Genomic prediction and allele mining of agronomic and morphological traits in pea (Pisum sativum) germplasm collections
Source: Front Plant Sci. 2023 Dec 22;14:1320506. doi: 10.3389/fpls.2023.1320506 (PMC10766761; doi:10.3389/fpls.2023.1320506)

Supplementary Figure 1. Quantile-Quantile plots of expected vs. observed association scores of 41,114 SNPs for nine qualitative traits. Different colors were attributed to SNPs basing on a classification in three categories depending on the ratio of SNP expected variances in different population strata (Milet et al., 2020), which were defined by DAPC cluster membership. The red line represents equality between the expected and observed quantiles and the grey area the associated 95% confidence interval.

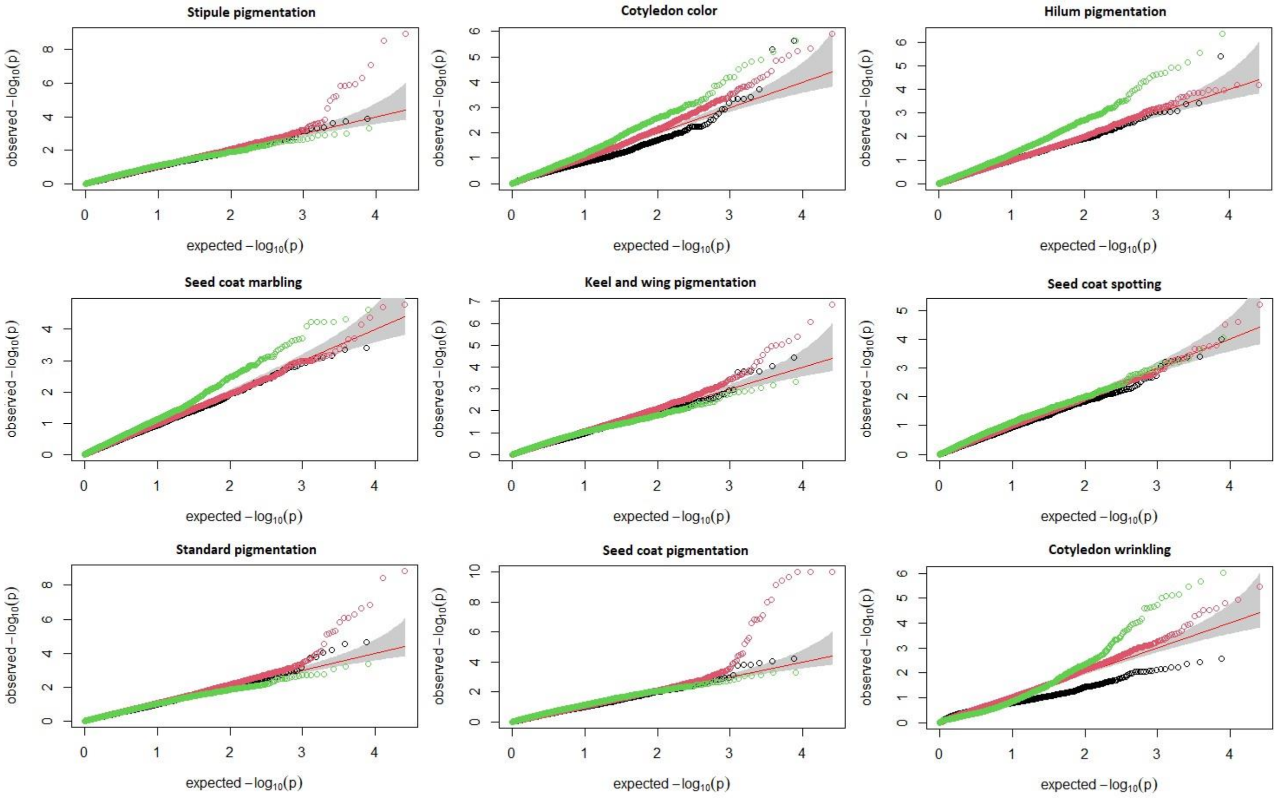

Supplement: Supplementary file 1 [file Image_1.pdf]
