# Supplementary material for: Genomic prediction and allele mining of agronomic and morphological traits in pea (Pisum sativum) germplasm collections
Source: Front Plant Sci. 2023 Dec 22;14:1320506. doi: 10.3389/fpls.2023.1320506 (PMC10766761; doi:10.3389/fpls.2023.1320506)

Supplementary Figure 3. Cross-entropy criterion for increasing K values based on the molecular data of 11,072 SNPs for a worldwide pea germplasm collection of 220 landraces from 19 regional pools and 11 modern cultivars.

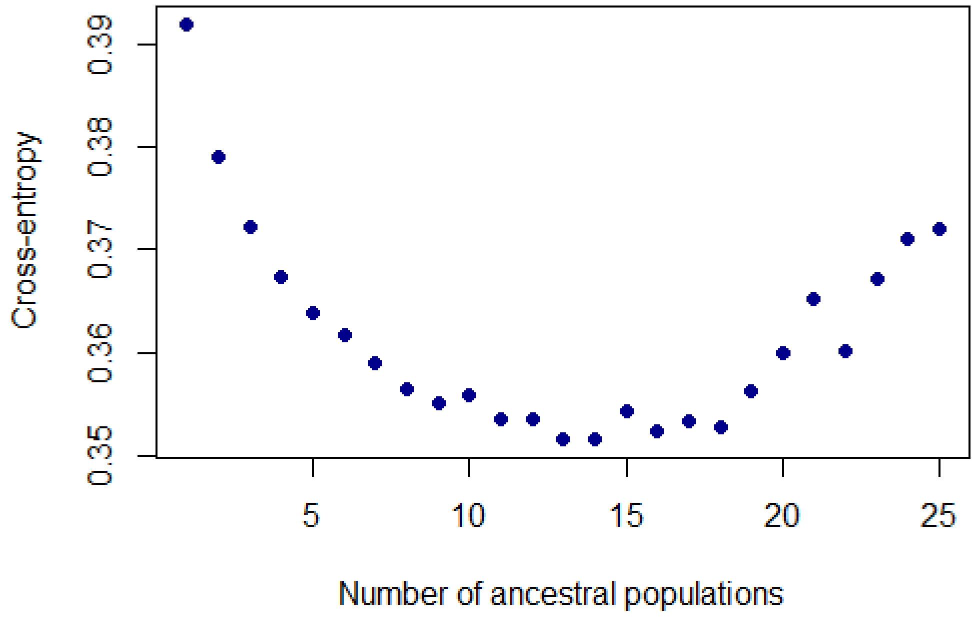

Supplement: Supplementary file 3 [file Image_3.pdf]
