# Supplementary material for: Genomic prediction and allele mining of agronomic and morphological traits in pea (Pisum sativum) germplasm collections
Source: Front Plant Sci. 2023 Dec 22;14:1320506. doi: 10.3389/fpls.2023.1320506 (PMC10766761; doi:10.3389/fpls.2023.1320506)

Supplementary Figure 4. Plots of linkage disequilibrium (r^2) decay with physical distance for pea chromosomes. r^2 was estimated on pairwise combinations of 41,114 SNPs within a 100 kb window for a worldwide germplasm collection of 220 landraces from 19 regional pools and 11 modern cultivars.

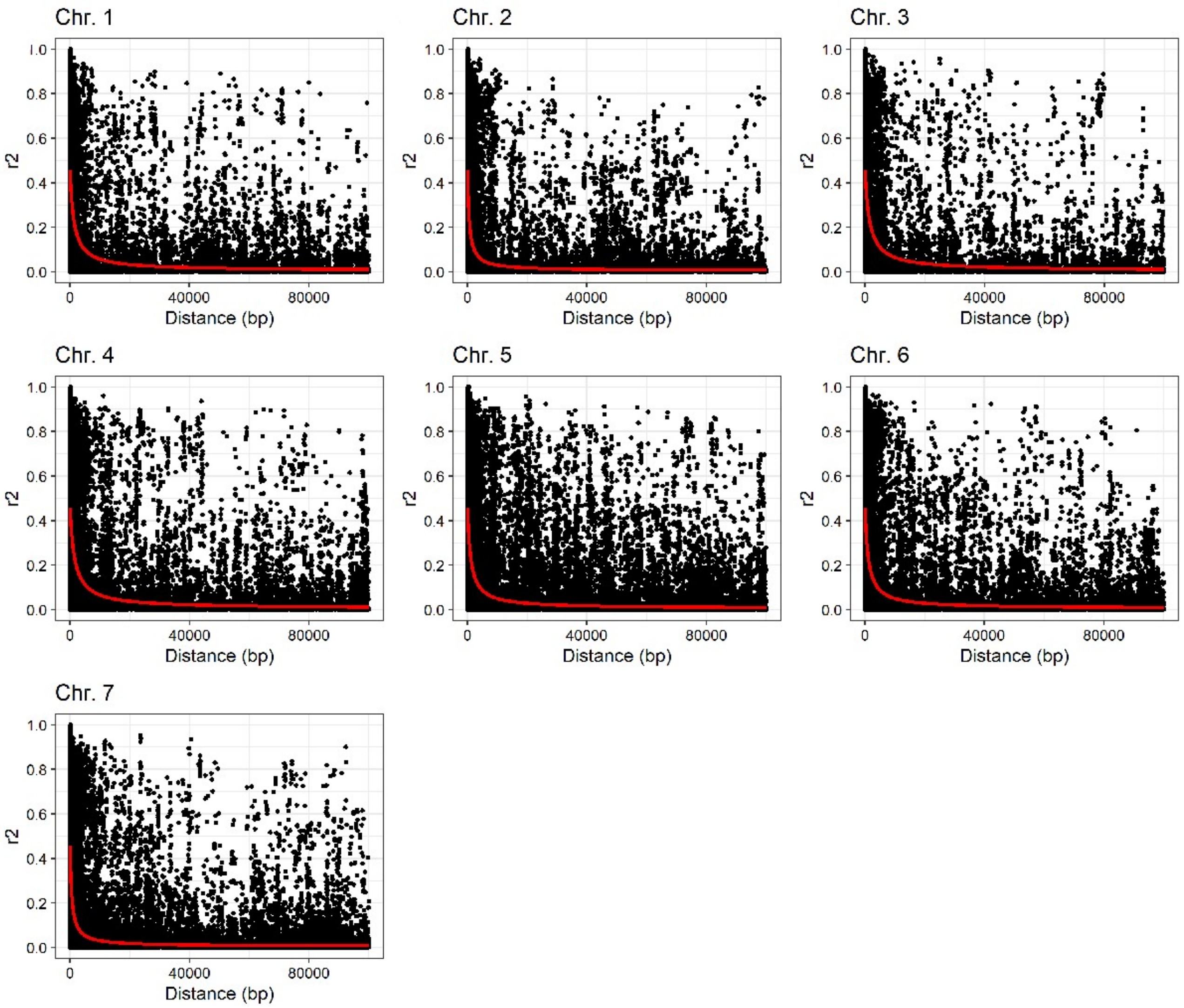

Supplement: Supplementary file 4 [file Image_4.pdf]
