# Supplementary material for: Genomic prediction and allele mining of agronomic and morphological traits in pea (Pisum sativum) germplasm collections
Source: Front Plant Sci. 2023 Dec 22;14:1320506. doi: 10.3389/fpls.2023.1320506 (PMC10766761; doi:10.3389/fpls.2023.1320506)

Supplementary Figure 6. Manhattan plots showing the non-significant association scores of 41,114 SNPs with two qualitative and one quantitative trait along pea chromosomes for a GWAS based on a worldwide germplasm collection of 220 landraces from 19 regional pools and 11 modern cultivars.

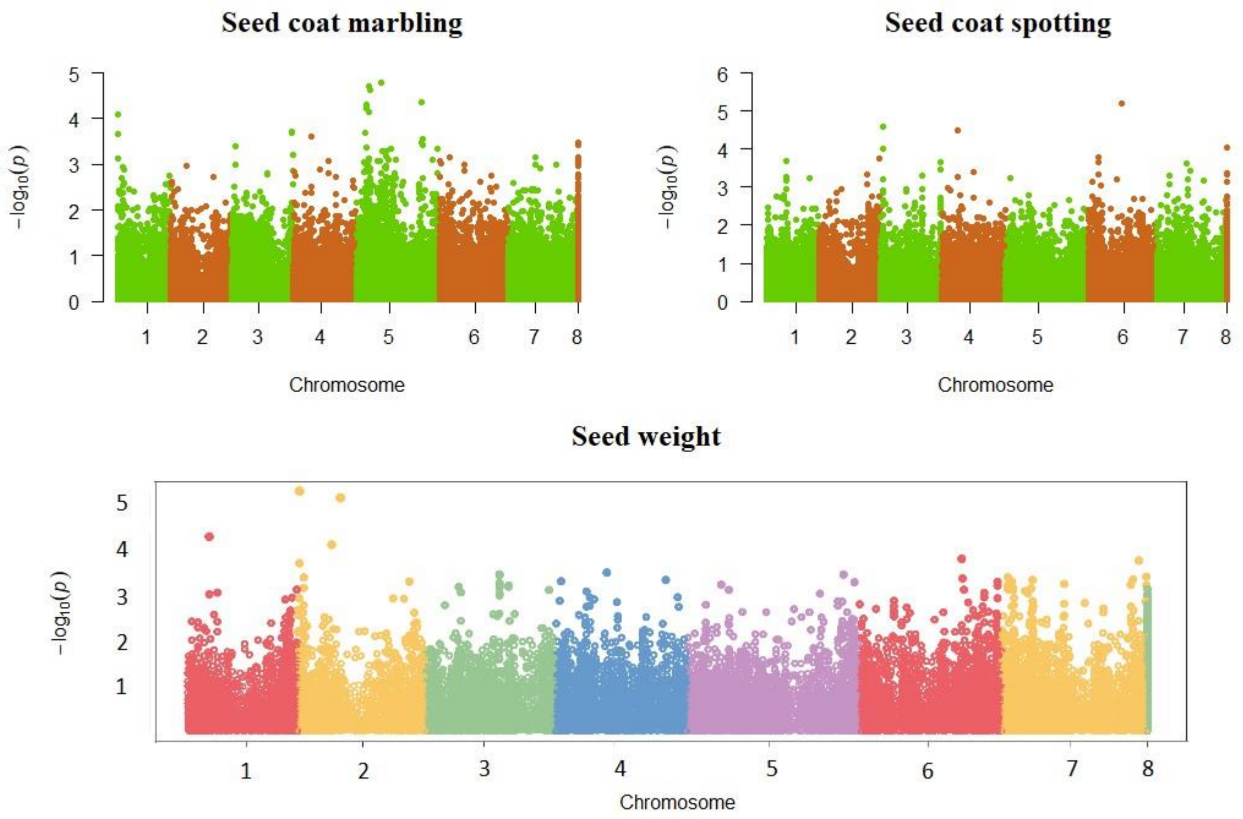

Supplement: Supplementary file 6 [file Image_6.pdf]
