# Supplementary material for: Genomic prediction and allele mining of agronomic and morphological traits in pea (Pisum sativum) germplasm collections
Source: Front Plant Sci. 2023 Dec 22;14:1320506. doi: 10.3389/fpls.2023.1320506 (PMC10766761; doi:10.3389/fpls.2023.1320506)
Supplement: Supplementary file 7 [file Table_1.pdf]

Supplementary Table 1. Accession name, germplasm pool, area of origin, and donor institution of a worldwide pea germplasm collection including 220 landraces from 19 regional pools and 11 modern cultivars.

Accession name	Germplasm pool	Geographic area	Germplasm type	Donor institution
IG116297	Turkey	Western Asia	Landrace/old cultivar	ICARDA
IG51989	India	India	Landrace/old cultivar	ICARDA
IG112140	Maghreb	Maghreb	Landrace/old cultivar	ICARDA
IG50673	Greece	Southern Europe	Landrace/old cultivar	ICARDA
IG123136	Ethiopia	Ethiopia	Landrace/old cultivar	ICARDA
IG50311	Turkey	Western Asia	Landrace/old cultivar	ICARDA
IG128863	Ukraine	Ukraine, Georgia, Russia	Landrace/old cultivar	ICARDA
IG51529	Ethiopia	Ethiopia	Landrace/old cultivar	ICARDA
IG51994	India	India	Landrace/old cultivar	ICARDA
IG49633	Greece	Southern Europe	Landrace/old cultivar	ICARDA
IG52455	Maghreb	Maghreb	Landrace/old cultivar	ICARDA
IG52596	Maghreb	Maghreb	Landrace/old cultivar	ICARDA
IG52459	Maghreb	Maghreb	Landrace/old cultivar	ICARDA
IG52036	China	China	Landrace/old cultivar	ICARDA
IG52442	Western Asia	Western Asia	Landrace/old cultivar	ICARDA
IG49610	Greece	Southern Europe	Landrace/old cultivar	ICARDA
IG123006	Ethiopia	Ethiopia	Landrace/old cultivar	ICARDA
IG123242	China	China	Landrace/old cultivar	ICARDA
IG50756	Greece	Southern Europe	Landrace/old cultivar	ICARDA
IG50358	Turkey	Western Asia	Landrace/old cultivar	ICARDA
IG50250	Turkey	Western Asia	Landrace/old cultivar	ICARDA
IG125543	Central Asia	Central Asia	Landrace/old cultivar	ICARDA

IG52383	China	China	Landrace/old cultivar	ICARDA
IG134619	Russia	Ukraine, Georgia, Russia	Landrace/old cultivar	ICARDA
IG134828	Georgia	Ukraine, Georgia, Russia	Landrace/old cultivar	ICARDA
IG123313	Maghreb	Maghreb	Landrace/old cultivar	ICARDA
IG50235	India	India	Landrace/old cultivar	ICARDA
IG115331	Nepal	Afghanistan, Nepal	Landrace/old cultivar	ICARDA
IG123237	China	China	Landrace/old cultivar	ICARDA
IG52535	Western Asia	Western Asia	Landrace/old cultivar	ICARDA
IG50559	Greece	Southern Europe	Landrace/old cultivar	ICARDA
IG52456	Maghreb	Maghreb	Landrace/old cultivar	ICARDA
IG52426	Western Asia	Western Asia	Landrace/old cultivar	ICARDA
IG123244	China	China	Landrace/old cultivar	ICARDA
IG125600	Central Asia	Central Asia	Landrace/old cultivar	ICARDA
IG116232	Turkey	Western Asia	Landrace/old cultivar	ICARDA
IG51948	India	India	Landrace/old cultivar	ICARDA
IG134080	Balkans	Central Europe	Landrace/old cultivar	ICARDA
IG49176	Ethiopia	Ethiopia	Landrace/old cultivar	ICARDA
IG123248	China	China	Landrace/old cultivar	ICARDA
IG49544	Georgia	Ukraine, Georgia, Russia	Landrace/old cultivar	ICARDA
IG51576	Ethiopia	Ethiopia	Landrace/old cultivar	ICARDA
IG123080	Western Asia	Western Asia	Landrace/old cultivar	ICARDA
IG51927	India	India	Landrace/old cultivar	ICARDA
IG134772	Ukraine	Ukraine, Georgia, Russia	Landrace/old cultivar	ICARDA
IG51687	Greece	Southern Europe	Landrace/old cultivar	ICARDA
IG115341	Nepal	Afghanistan, Nepal	Landrace/old cultivar	ICARDA

IG50570	Greece	Southern Europe	Landrace/old cultivar	ICARDA
IG114914	Nepal	Afghanistan, Nepal	Landrace/old cultivar	ICARDA
IG50584	Greece	Southern Europe	Landrace/old cultivar	ICARDA
IG123245	China	China	Landrace/old cultivar	ICARDA
IG52586	Maghreb	Maghreb	Landrace/old cultivar	ICARDA
IG134788	Russia	Ukraine, Georgia, Russia	Landrace/old cultivar	ICARDA
IG125597	Central Asia	Central Asia	Landrace/old cultivar	ICARDA
IG122966	Central Asia	Central Asia	Landrace/old cultivar	ICARDA
IG134718	Georgia	Ukraine, Georgia, Russia	Landrace/old cultivar	ICARDA
IG52496	Turkey	Western Asia	Landrace/old cultivar	ICARDA
IG115145	Nepal	Afghanistan, Nepal	Landrace/old cultivar	ICARDA
IG123118	Ethiopia	Ethiopia	Landrace/old cultivar	ICARDA
IG52040	China	China	Landrace/old cultivar	ICARDA
IG123041	Central Asia	Central Asia	Landrace/old cultivar	ICARDA
IG49181	Afghanistan	Afghanistan, Nepal	Landrace/old cultivar	ICARDA
IG52417	Western Asia	Western Asia	Landrace/old cultivar	ICARDA
IG51957	India	India	Landrace/old cultivar	ICARDA
IG114899	Nepal	Afghanistan, Nepal	Landrace/old cultivar	ICARDA
IG134746	Ukraine	Ukraine, Georgia, Russia	Landrace/old cultivar	ICARDA
IG115114	Nepal	Afghanistan, Nepal	Landrace/old cultivar	ICARDA
IG125324	Russia	Ukraine, Georgia, Russia	Landrace/old cultivar	ICARDA
IG50303	India	India	Landrace/old cultivar	ICARDA
IG50641	Greece	Southern Europe	Landrace/old cultivar	ICARDA
IG128856	Georgia	Ukraine, Georgia, Russia	Landrace/old cultivar	ICARDA
IG125471	Georgia	Ukraine, Georgia, Russia	Landrace/old cultivar	ICARDA

IG134109	Balkans	Central Europe	Landrace/old cultivar	ICARDA
IG49189	Afghanistan	Afghanistan, Nepal	Landrace/old cultivar	ICARDA
IG124857	Afghanistan	Afghanistan, Nepal	Landrace/old cultivar	ICARDA
IG51520	Ethiopia	Ethiopia	Landrace/old cultivar	ICARDA
IG134862	Balkans	Central Europe	Landrace/old cultivar	ICARDA
IG115100	Nepal	Afghanistan, Nepal	Landrace/old cultivar	ICARDA
IG125326	Russia	Ukraine, Georgia, Russia	Landrace/old cultivar	ICARDA
IG114977	Nepal	Afghanistan, Nepal	Landrace/old cultivar	ICARDA
IG124664	China	China	Landrace/old cultivar	ICARDA
IG52050	India	India	Landrace/old cultivar	ICARDA
IG122974	Central Asia	Central Asia	Landrace/old cultivar	ICARDA
IG122996	Central Asia	Central Asia	Landrace/old cultivar	ICARDA
IG51976	India	India	Landrace/old cultivar	ICARDA
IG115266	Nepal	Afghanistan, Nepal	Landrace/old cultivar	ICARDA
IG50362	Turkey	Western Asia	Landrace/old cultivar	ICARDA
IG134744	Ukraine	Ukraine, Georgia, Russia	Landrace/old cultivar	ICARDA
IG123312	Central Asia	Central Asia	Landrace/old cultivar	ICARDA
IG134094	Balkans	Central Europe	Landrace/old cultivar	ICARDA
IG134841	Balkans	Central Europe	Landrace/old cultivar	ICARDA
IG50669	China	China	Landrace/old cultivar	ICARDA
IG115228	Nepal	Afghanistan, Nepal	Landrace/old cultivar	ICARDA
IG128973	Balkans	Central Europe	Landrace/old cultivar	ICARDA
IG125415	Russia	Ukraine, Georgia, Russia	Landrace/old cultivar	ICARDA
IG125550	China	China	Landrace/old cultivar	ICARDA
IG125336	Ukraine	Ukraine, Georgia, Russia	Landrace/old cultivar	ICARDA

IG125472	Georgia	Ukraine, Georgia, Russia	Landrace/old cultivar	ICARDA
IG123073	Maghreb	Maghreb	Landrace/old cultivar	ICARDA
IG134823	Russia	Ukraine, Georgia, Russia	Landrace/old cultivar	ICARDA
IG123240	China	China	Landrace/old cultivar	ICARDA
IG125589	Central Asia	Central Asia	Landrace/old cultivar	ICARDA
IG134707	Georgia	Ukraine, Georgia, Russia	Landrace/old cultivar	ICARDA
IG129002	Greece	Southern Europe	Landrace/old cultivar	ICARDA
IG52534	Western Asia	Western Asia	Landrace/old cultivar	ICARDA
IG123311	Central Asia	Central Asia	Landrace/old cultivar	ICARDA
IG123050	Turkey	Western Asia	Landrace/old cultivar	ICARDA
IG134060	Balkans	Central Europe	Landrace/old cultivar	ICARDA
IG134609	Georgia	Ukraine, Georgia, Russia	Landrace/old cultivar	ICARDA
IG123227	China	China	Landrace/old cultivar	ICARDA
IG51513	Ethiopia	Ethiopia	Landrace/old cultivar	ICARDA
IG52521	Turkey	Western Asia	Landrace/old cultivar	ICARDA
IG50935	Greece	Southern Europe	Landrace/old cultivar	ICARDA
IG50357	Turkey	Western Asia	Landrace/old cultivar	ICARDA
IG123211	China	China	Landrace/old cultivar	ICARDA
IG123028	Afghanistan	Afghanistan, Nepal	Landrace/old cultivar	ICARDA
IG51891	India	India	Landrace/old cultivar	ICARDA
IG51562	Ethiopia	Ethiopia	Landrace/old cultivar	ICARDA
IG123034	Afghanistan	Afghanistan, Nepal	Landrace/old cultivar	ICARDA
IG128913	Ukraine	Ukraine, Georgia, Russia	Landrace/old cultivar	ICARDA
IG123029	Afghanistan	Afghanistan, Nepal	Landrace/old cultivar	ICARDA
IG134770	Ukraine	Ukraine, Georgia, Russia	Landrace/old cultivar	ICARDA

IG128934	Ukraine	Ukraine, Georgia, Russia	Landrace/old cultivar	ICARDA
IG123004	Maghreb	Maghreb	Landrace/old cultivar	ICARDA
IG134857	Balkans	Central Europe	Landrace/old cultivar	ICARDA
IG134782	Russia	Ukraine, Georgia, Russia	Landrace/old cultivar	ICARDA
IG123280	Central Asia	Central Asia	Landrace/old cultivar	ICARDA
IG123021	Afghanistan	Afghanistan, Nepal	Landrace/old cultivar	ICARDA
IG51991	India	India	Landrace/old cultivar	ICARDA
IG51551	Ethiopia	Ethiopia	Landrace/old cultivar	ICARDA
IG128887	Russia	Ukraine, Georgia, Russia	Landrace/old cultivar	ICARDA
IG134621	Russia	Ukraine, Georgia, Russia	Landrace/old cultivar	ICARDA
IG52401	Western Asia	Western Asia	Landrace/old cultivar	ICARDA
IG52081	Ethiopia	Ethiopia	Landrace/old cultivar	ICARDA
IG51688	Greece	Southern Europe	Landrace/old cultivar	ICARDA
IG125378	Russia	Ukraine, Georgia, Russia	Landrace/old cultivar	ICARDA
IG128983	Russia	Ukraine, Georgia, Russia	Landrace/old cultivar	ICARDA
IG51536	Ethiopia	Ethiopia	Landrace/old cultivar	ICARDA
IG123288	Central Asia	Central Asia	Landrace/old cultivar	ICARDA
IG51516	Ethiopia	Ethiopia	Landrace/old cultivar	ICARDA
IG50248	Turkey	Western Asia	Landrace/old cultivar	ICARDA
IG52005	India	India	Landrace/old cultivar	ICARDA
IG124843	Ukraine	Ukraine, Georgia, Russia	Landrace/old cultivar	ICARDA
IG52017	India	India	Landrace/old cultivar	ICARDA
IG123102	Ethiopia	Ethiopia	Landrace/old cultivar	ICARDA
IG123281	Central Asia	Central Asia	Landrace/old cultivar	ICARDA
IG50592	China	China	Landrace/old cultivar	ICARDA

IG52092	Ethiopia	Ethiopia	Landrace/old cultivar	ICARDA
IG51993	India	India	Landrace/old cultivar	ICARDA
IG134649	Ukraine	Ukraine, Georgia, Russia	Landrace/old cultivar	ICARDA
IG49224	Georgia	Ukraine, Georgia, Russia	Landrace/old cultivar	ICARDA
IG125469	Georgia	Ukraine, Georgia, Russia	Landrace/old cultivar	ICARDA
IG52367	Afghanistan	Afghanistan, Nepal	Landrace/old cultivar	ICARDA
IG134870	Balkans	Central Europe	Landrace/old cultivar	ICARDA
IG116455	Turkey	Western Asia	Landrace/old cultivar	ICARDA
IG49327	Greece	Southern Europe	Landrace/old cultivar	ICARDA
IG134750	Ukraine	Ukraine, Georgia, Russia	Landrace/old cultivar	ICARDA
IG49203	Afghanistan	Afghanistan, Nepal	Landrace/old cultivar	ICARDA
IG52595	Maghreb	Maghreb	Landrace/old cultivar	ICARDA
MG 100948	Italy	Southern Europe	Landrace/old cultivar	CNR-IBBR, Bari
MG 101126	Italy	Southern Europe	Landrace/old cultivar	CNR-IBBR, Bari
MG 106069	Italy	Southern Europe	Landrace/old cultivar	CNR-IBBR, Bari
MG 106871	Italy	Southern Europe	Landrace/old cultivar	CNR-IBBR, Bari
MG 110243	Italy	Southern Europe	Landrace/old cultivar	CNR-IBBR, Bari
MG 110416	Italy	Southern Europe	Landrace/old cultivar	CNR-IBBR, Bari
MG 110417	Italy	Southern Europe	Landrace/old cultivar	CNR-IBBR, Bari
MG 110418	Italy	Southern Europe	Landrace/old cultivar	CNR-IBBR, Bari
MG 111850	Italy	Southern Europe	Landrace/old cultivar	CNR-IBBR, Bari
MG 111988	Italy	Southern Europe	Landrace/old cultivar	CNR-IBBR, Bari
MG 115084	Italy	Southern Europe	Landrace/old cultivar	CNR-IBBR, Bari
ZP0064	Spain	Southern Europe	Landrace/old cultivar	ITACyL Valladolid
ZP0076	Spain	Southern Europe	Landrace/old cultivar	ITACyL Valladolid

ZP0126	Spain	Southern Europe	Landrace/old cultivar	ITACyL Valladolid
ZP0181	Spain	Southern Europe	Landrace/old cultivar	ITACyL Valladolid
ZP0202	Spain	Southern Europe	Landrace/old cultivar	ITACyL Valladolid
ZP0213	Spain	Southern Europe	Landrace/old cultivar	ITACyL Valladolid
ZP0535	Spain	Southern Europe	Landrace/old cultivar	ITACyL Valladolid
ZP0798	Spain	Southern Europe	Landrace/old cultivar	ITACyL Valladolid
ZP0799	Spain	Southern Europe	Landrace/old cultivar	ITACyL Valladolid
ZP1261	Spain	Southern Europe	Landrace/old cultivar	ITACyL Valladolid
ZP1264	Spain	Southern Europe	Landrace/old cultivar	ITACyL Valladolid
ZP1282	Spain	Southern Europe	Landrace/old cultivar	ITACyL Valladolid
ZP1294	Spain	Southern Europe	Landrace/old cultivar	ITACyL Valladolid
ZP1300	Spain	Southern Europe	Landrace/old cultivar	ITACyL Valladolid
Witham Wonder	United Kingdom	Western Europe	Landrace/old cultivar	JIC Norwich
Emerald Gem	United Kingdom	Western Europe	Landrace/old cultivar	JIC Norwich
Englishsabel	United Kingdom	Western Europe	Landrace/old cultivar	JIC Norwich
Fillbasket	United Kingdom	Western Europe	Landrace/old cultivar	JIC Norwich
English Wonder	United Kingdom	Western Europe	Landrace/old cultivar	JIC Norwich
Kentish Invicta	United Kingdom	Western Europe	Landrace/old cultivar	JIC Norwich
Alderman	United Kingdom	Western Europe	Landrace/old cultivar	JIC Norwich
Mummy Pea	United Kingdom	Western Europe	Landrace/old cultivar	JIC Norwich
Magnum Bonum	United Kingdom	Western Europe	Landrace/old cultivar	JIC Norwich
Raina Victoria	United Kingdom	Western Europe	Landrace/old cultivar	JIC Norwich
Knights Marrow	United Kingdom	Western Europe	Landrace/old cultivar	JIC Norwich
Knights Dwarf White	United Kingdom	Western Europe	Landrace/old cultivar	JIC Norwich
English Maple	United Kingdom	Western Europe	Landrace/old cultivar	JIC Norwich

Gloire de Correze	France	Western Europe	Landrace/old cultivar	INRA Dijon
Serpette D'Auvergne	France	Western Europe	Landrace/old cultivar	INRA Dijon
Picar	France	Western Europe	Landrace/old cultivar	INRA Dijon
Quarante-deux de Sarcelles	France	Western Europe	Landrace/old cultivar	INRA Dijon
Piver	France	Western Europe	Landrace/old cultivar	INRA Dijon
Triomphe de Roissy	France	Western Europe	Landrace/old cultivar	INRA Dijon
Chemin Long	France	Western Europe	Landrace/old cultivar	INRA Dijon
Serpette de Vitry	France	Western Europe	Landrace/old cultivar	INRA Dijon
Gris de Bourgogne	France	Western Europe	Landrace/old cultivar	INRA Dijon
Haute Loire	France	Western Europe	Landrace/old cultivar	INRA Dijon
Champagne	France	Western Europe	Landrace/old cultivar	INRA Dijon
Cote D'Or	France	Western Europe	Landrace/old cultivar	INRA Dijon
Serpette de Paris	France	Western Europe	Landrace/old cultivar	INRA Dijon
CL 19cvs1	Central Europe	Central Europe	Landrace/old cultivar	CRI, Praha
Hrach Z Pardubic	Central Europe	Central Europe	Landrace/old cultivar	CRI, Praha
Kocovska 108	Central Europe	Central Europe	Landrace/old cultivar	CRI, Praha
Pulawska Slodka Nr 2	Central Europe	Central Europe	Landrace/old cultivar	IPK Gatersleben
Kapucin Belokvety	Central Europe	Central Europe	Landrace/old cultivar	CRI, Praha
Landrace Orava	Central Europe	Central Europe	Landrace/old cultivar	CRI, Praha
PIS 278	Central Europe	Central Europe	Landrace/old cultivar	IPK Gatersleben
PIS 657	Central Europe	Central Europe	Landrace/old cultivar	IPK Gatersleben
PIS 845	Central Europe	Central Europe	Landrace/old cultivar	IPK Gatersleben
PIS 2856	Central Europe	Central Europe	Landrace/old cultivar	IPK Gatersleben
Attika	Improved Variety	Improved Variety	Improved Variety	-
Genial	Improved Variety	Improved Variety	Improved Variety	-

Messire	Improved Variety	Improved Variety	Improved Variety	-
Santana	Improved Variety	Improved Variety	Improved Variety	-
Spirale	Improved Variety	Improved Variety	Improved Variety	-
Cartuce	Improved Variety	Improved Variety	Improved Variety	-
Dove	Improved Variety	Improved Variety	Improved Variety	-
Enduro	Improved Variety	Improved Variety	Improved Variety	-
Isard	Improved Variety	Improved Variety	Improved Variety	-
Viriato	Improved Variety	Improved Variety	Improved Variety	-
Cigarron	Improved Variety	Improved Variety	Improved Variety	-
