# Supplementary material for: Genomic prediction and allele mining of agronomic and morphological traits in pea (Pisum sativum) germplasm collections
Source: Front Plant Sci. 2023 Dec 22;14:1320506. doi: 10.3389/fpls.2023.1320506 (PMC10766761; doi:10.3389/fpls.2023.1320506)
Supplement: Supplementary file 8 [file Table_2.pdf]

Supplementary Table 2. Significant markers detected by a GWAS based on 41,114 SNPs and performed on a worldwide pea germplasm collection of 220 landraces from 19 regional pools and 11 modern cultivars for seven qualitative and four quantitative traits. The SNPs are presented from the most to the least significant for each trait with the relative MAF and estimated effect.

SNP	Trait	MAF	Effect
chr6LG2_68307452	Stipule pigmentation	0.38	1.84
chr6LG2_68265355	Stipule pigmentation	0.37	1.87
chr6LG2_67835918	Stipule pigmentation	0.43	1.60
chr6LG2_235650075	Stipule pigmentation	0.46	1.49
chr6LG2_27407716	Stipule pigmentation	0.25	1.77
chr1LG6_356439618	Hilum pigmentation	0.14	2.15
chr6LG2_68307452	Keel and wing pigmentation	0.49	1.43
chr6LG2_68265355	Keel and wing pigmentation	0.49	1.35
chr6LG2_68307452	Standard pigmentation	0.49	1.48
chr6LG2_68265355	Standard pigmentation	0.49	1.47
chr6LG2_68261112	Standard pigmentation	0.50	1.34
chr6LG2_67641787	Standard pigmentation	0.41	1.35
chr6LG2_67835918	Standard pigmentation	0.45	1.22
chr6LG2_67836000	Standard pigmentation	0.47	1.21
chr6LG2_235650075	Standard pigmentation	0.43	1.17
chr6LG2_68265355	Seed coat pigmentation	0.50	1.97
chr6LG2_68261112	Seed coat pigmentation	0.50	2.00
chr6LG2_68307452	Seed coat pigmentation	0.50	1.90
chr6LG2_67835918	Seed coat pigmentation	0.43	1.77
chr6LG2_235650004	Seed coat pigmentation	0.44	1.87
chr6LG2_67641787	Seed coat pigmentation	0.40	1.88
chr6LG2_67836000	Seed coat pigmentation	0.45	1.65
chr6LG2_235650075	Seed coat pigmentation	0.41	1.60
chr6LG2_235738439	Seed coat pigmentation	0.46	1.55
chr6LG2_68270289	Seed coat pigmentation	0.40	1.49
chr6LG2_68270246	Seed coat pigmentation	0.41	1.47
chr6LG2_68270304	Seed coat pigmentation	0.39	1.48
chr6LG2_235739982	Seed coat pigmentation	0.43	1.57
chr3LG5_65566666	Cotyledon wrinkling	0.07	2.68
scaffold01735_103693	Onset of flowering	0.34	1.61
chr5LG3_507674783	Onset of flowering	0.49	1.04
chr4LG4_252131852	Onset of flowering	0.13	-1.39
chr6LG2_374770044	Onset of flowering	0.06	2.26
chr6LG2_72901872	Straw yield	0.06	0.56
scaffold00384_33569	Straw yield	0.24	-0.28
chr6LG2_72901872	Grain yield	0.06	0.32
chr1LG6_47025851	Grain yield	0.15	0.22
chr7LG7_183744462	Grain yield	0.13	-0.27
chr4LG4_186752146	Grain yield	0.14	-0.34
chr5LG3_492526140	Protein content	0.10	0.95
