# Supplementary material for: Genomic prediction and allele mining of agronomic and morphological traits in pea (Pisum sativum) germplasm collections
Source: Front Plant Sci. 2023 Dec 22;14:1320506. doi: 10.3389/fpls.2023.1320506 (PMC10766761; doi:10.3389/fpls.2023.1320506)
Supplement: Supplementary file 9 [file Table_3.pdf]

Supplementary Table 3. List of genes potentially associated to the significant SNPs detected by a GWAS based on 41,114 SNPs and performed on a worldwide pea germplasm collection of 220 landraces from 19 regional pools and 11 modern cultivars for seven qualitative and four quantitative traits. Candidate genes were identified either by scanning a region as long as the average chromosome distance at which LD dropped to 0.05 in both directions from each significant SNP, or by verifying SNP linkage with previously cloned loci, and are reported with their annotated function (https://urgi.versailles.inra.fr/). For qualitative traits, the genomic region surrounding the most significant SNP in each peak was scanned for candidate gene research.

SNP	Scanned interval	Trait	Gene	Cloned	Function
chr6LG2_27407716	chr6LG2:27397583..27417849	Stipule pigmentation	Psat6g034040	no	Flavin-binding monooxygenase-like
chr6LG2_235650075	chr6LG2:235639871..235660137	Stipule and standard pigmentation	Psat6g125800	no	Xylanase inhibitor C-terminal
chr6LG2_68307452	-	Stipule, standard, and keel and wing pigmentation	Psat6g060480	yes	BHLH-MYC and R2R3-MYB transcription factors N-terminal
chr1LG6_356439618	-	Hilum pigmentation	Psat1g206360	yes	Protein of unknown function (DUF_B2219)
chr6LG2_68265355	-	Seed coat pigmentation	Psat6g060480	yes	BHLH-MYC and R2R3-MYB transcription factors N-terminal
chr6LG2_235650004	chr6LG2:235639871..235660137	Seed coat pigmentation	Psat6g125800	no	Xylanase inhibitor C-terminal
chr3LG5_65566666	-	Cotyledon wrinkling	Psat3g034640	yes	Alpha amylase + C-terminal all-beta domain
scaffold01735_103693	-	Onset of flowering	Psat0s1735g0240	no	ZIP Zinc transporter
chr5LG3_507674783	chr5LG3:507664733..507684833	Onset of flowering	Psat5g255760	no	NA
chr4LG4_252131852	chr4LG4:252117658..252146046	Onset of flowering	Psat4g132240	no	Alpha-amylase C-terminal beta-sheet domain
chr6LG2_374770044	chr6LG2:374759911..374780177	Onset of flowering	Psat6g186000	no	Unknown gene

chr6LG2_374770044	chr6LG2:374759911..374780177	Onset of flowering	Psat6g186040	no	Protein of unknown function (DUF410)
chr6LG2_72901872	chr6LG2:72891739..72912005	Straw yield; grain yield	Psat6g064800	no	Helix-loop-helix DNA-binding domain
scaffold00384_33569	-	Straw yield	NA	no	NA
chr1LG6_47025851	chr1LG6:47013992..47037710	Grain yield	Psat1g031400	no	Protein kinase domain
chr4LG4_186752146	chr4LG4:186737952..186766340	Grain yield	Psat4g098400	no	RNA recognition motif. (a.k.a. RRM + RBD + or RNP domain)
chr7LG7_183744462	chr7LG7:183738959..183749965	Grain yield	Psat7g111400	no	No apical meristem (NAM) protein
chr5LG3_492526140	chr5LG3:492516090..492536190	Protein content	Psat5g246720	no	Rhomboid family
