# Supplementary material for: Genomic prediction and allele mining of agronomic and morphological traits in pea (Pisum sativum) germplasm collections
Source: Front Plant Sci. 2023 Dec 22;14:1320506. doi: 10.3389/fpls.2023.1320506 (PMC10766761; doi:10.3389/fpls.2023.1320506)
Supplement: Supplementary file 10 [file Table_4.pdf]

Supplementary Table 4. Linkage disequilibrium (r^2) between pairwise combinations of significant SNPs according to Bonferroni threshold at 5% and, if not included, the first SNP on the opposite side of the cloned gene for each of seven pea qualitative traits (flower pigmentation includes SNPs for both standard pigmentation and keel and wing pigmentation). Although not significant, the SNP featuring the lowest *p* value for cotyledon color was included due to its proximity to the significance threshold. Significant SNPs were identified by a GWAS based on 41,114 SNPs performed on a worldwide germplasm collection of 220 landraces from 19 regional pools and 11 modern cultivars.

Stipule pigmentation

	chr6LG2_68370459	chr6LG2_68307452	chr6LG2_68265355	chr6LG2_67835918	chr6LG2_235650075	chr6LG2_27407716
chr6LG2_68370459	1	0.07	0.08	0.08	0	0
chr6LG2_68307452	0.07	1	0.93	0.62	0.39	0.06
chr6LG2_68265355	0.08	0.93	1	0.65	0.39	0.06
chr6LG2_67835918	0.08	0.62	0.65	1	0.29	0.07
chr6LG2_235650075	0	0.39	0.39	0.29	1	0.11
chr6LG2_27407716	0	0.06	0.06	0.07	0.11	1

Cotyledon color

	chr2LG1_419560365	chr2LG1_424229115
chr2LG1_419560365	1	0.05
chr2LG1_424229115	0.05	1

Hilum pigmentation

	chr1LG6_356439618	chr1LG6_356809768
chr1LG6_356	1	0.10

439618		
chr1LG6_356809768	0.10	1

Flower pigmentation

	chr6LG2_68370459	chr6LG2_68307452	chr6LG2_68265355	chr6LG2_68261112	chr6LG2_67641787	chr6LG2_67835918	chr6LG2_67836000	chr6LG2_235650075
chr6LG2_68370459	1	0.07	0.08	0.06	0.03	0.08	0.05	0
chr6LG2_68307452	0.07	1	0.93	0.72	0.69	0.62	0.61	0.39
chr6LG2_68265355	0.08	0.93	1	0.79	0.71	0.65	0.65	0.39
chr6LG2_68261112	0.06	0.72	0.79	1	0.58	0.50	0.51	0.33
chr6LG2_67641787	0.03	0.69	0.71	0.58	1	0.49	0.48	0.34
chr6LG2_67835918	0.08	0.62	0.65	0.50	0.49	1	0.88	0.29
chr6LG2_67836000	0.05	0.61	0.65	0.51	0.48	0.88	1	0.32
chr6LG2_235650075	0	0.39	0.39	0.33	0.34	0.29	0.32	1

Seed coat pigmentation

	chr6LG2_235739982	chr6LG2_68370459	chr6LG2_68307452	chr6LG2_68265355	chr6LG2_68261112	chr6LG2_67641787	chr6LG2_67835918	chr6LG2_67836000	chr6LG2_235650075	chr6LG2_235650004	chr6LG2_68270246	chr6LG2_68270289	chr6LG2_235738439	chr6LG2_68270304
chr6LG2_235739982	1	0.01	0.41	0.43	0.37	0.40	0.36	0.31	0.23	0.38	0.17	0.18	0.64	0.17
chr6LG2_68370459	0.01	1	0.07	0.08	0.06	0.03	0.08	0.05	0	0	0.11	0.11	0.05	0.12
chr6LG2_68307452	0.41	0.07	1	0.93	0.72	0.69	0.62	0.61	0.39	0.51	0.51	0.51	0.49	0.50
chr6LG2_68265355	0.43	0.08	0.93	1	0.79	0.71	0.65	0.65	0.39	0.51	0.57	0.57	0.49	0.56
chr6LG2_68261112	0.37	0.06	0.72	0.79	1	0.58	0.50	0.51	0.33	0.45	0.48	0.47	0.44	0.46

chr6LG2_67641787	0.40	0.03	0.69	0.71	0.58	1	0.49	0.48	0.34	0.39	0.47	0.47	0.49	0.47
chr6LG2_67835918	0.36	0.08	0.62	0.65	0.50	0.49	1	0.88	0.29	0.41	0.37	0.37	0.43	0.37
chr6LG2_67836000	0.31	0.05	0.61	0.65	0.51	0.48	0.88	1	0.32	0.42	0.40	0.40	0.39	0.39
chr6LG2_235650075	0.23	0	0.39	0.39	0.33	0.34	0.29	0.32	1	0.49	0.34	0.33	0.30	0.31
chr6LG2_235650004	0.38	0	0.51	0.51	0.45	0.39	0.41	0.42	0.49	1	0.35	0.32	0.48	0.31
chr6LG2_68270246	0.17	0.11	0.51	0.57	0.48	0.47	0.37	0.40	0.34	0.35	1	0.94	0.24	0.94
chr6LG2_68270289	0.18	0.11	0.51	0.57	0.47	0.47	0.37	0.40	0.33	0.32	0.94	1	0.24	0.96
chr6LG2_235738439	0.64	0.05	0.49	0.49	0.44	0.49	0.43	0.39	0.30	0.48	0.24	0.24	1	0.23
chr6LG2_68270304	0.17	0.12	0.50	0.56	0.46	0.47	0.37	0.39	0.31	0.31	0.94	0.96	0.23	1

Cotyledon wrinkling

	chr3LG5_65566666	chr3LG5_71670671
chr3LG5_65566666	1	0.30
chr3LG5_71670671	0.30	1
